# Shared immune perturbations in hepatocellular carcinoma-depression comorbidity and synergistic immunotherapeutic effects of fluoxetine combined with anti-PD-1

**DOI:** 10.3389/fimmu.2026.1909503

**Published:** 2026-09-25

**Authors:** Shanshan Wang, Yunwei Gao, Qianbin Li, Huimin Song, Mei Ping, Honghong Shen, Tong Zhao, Yingli Wang, Rong Zhang

**Affiliations:** 1 Department of Basic Medical Sciences, Shanxi University of Chinese Medicine, Jinzhong, Shanxi, China; 2 State Key Laboratory of Traditional Chinese Medicine Syndrome, Zhuhai, Guangdong, China; 3 Guangdong Provincial Key Laboratory of Translational Cancer Research of Chinese Medicines, International Institute for Translational Chinese Medicine, School of Pharmaceutical Science, Guangzhou University of Chinese Medicine, Guangzhou, Guangdong, China; 4 The Affiliated Hospital of Shanxi University of Chinese Medicine, Taiyuan, Shanxi, China; 5 Department of Gastrointestinal Oncology, The First Hospital of Shanxi Medical University, Taiyuan, Shanxi, China; 6 Department of Pathology, The Second Hospital of Shanxi Medical University, Taiyuan, Shanxi, China

**Keywords:** immune dysregulation, immunometabolism, liver cancer-depression comorbidity, multi-omics, tryptophan metabolism

## Abstract

**Background:**

Hepatocellular carcinoma (HCC) often coexists with depression, elevating patient mortality by 39%. The tryptophan metabolic axis bridges tumor and neuropsychiatric immune dysfunction, yet the immunometabolic crosstalk underlying HCC-depression comorbidity remains poorly defined, and reliable combinatorial immunotherapies are absent.

**Methods:**

We integrated public multi-omics datasets including TCGA, GEO, eQTLGen and FinnGen, and performed two-sample MR as well as genetic colocalization analyses to screen hub genes associated with HCC-depression comorbidity. GSEA/GSVA, single-cell and spatial transcriptomics were adopted to dissect gene expression and perturbed tryptophan immune pathways. In silico docking predicted fluoxetine binding to BTN3A3, GLCE and RBCK1. A Hepa1-6/CUMS comorbid mouse model combined with targeted tryptophan LC-MS metabolomics, flow cytometry and ELISA was used to verify combined therapeutic effects and tissue molecular alterations.

**Results:**

Seven overlapping risk genes for HCC and depression were identified. *BTN3A3*, *GLCE* and *RBCK1* reached strict colocalization thresholds (SNP.PP.H4 > 0.90) and were predominantly expressed in T cells and M2 macrophages linked to tryptophan metabolism. Fluoxetine displayed stable binding affinity with all three hub proteins via molecular simulation. *In vivo* assays proved fluoxetine plus anti-PD-1 restrained tumor progression, alleviated depression-like phenotypes, normalized tissue GLCE/RBCK1 levels and restored disordered tryptophan metabolism and immunosuppressive microenvironment in comorbid mice.

**Conclusions:**

This work characterizes tryptophan-mediated immune disturbance in HCC-depression comorbidity. *GLCE* and *RBCK1* correlate with comorbid immunometabolic disorders. Fluoxetine combined with anti-PD-1 exerts synergistic immunoregulatory effects by reshaping tryptophan-related immune homeostasis, offering a preclinical metabolic immunotherapy strategy for concurrent HCC and depression.

## 1 Background

According to the 2024 report from the International Agency for Research on Cancer (IARC) of the World Health Organization, approximately 21.2% of cancer patients worldwide suffer from depression, with the incidence rates of depression and anxiety among liver cancer patients reaching 20.7% (1, 2).However, approximately three-quarters of cancer patients with prominent depressive symptoms have never undergone any psychological assessment or pharmacotherapeutic intervention. Thus, the management of cancer complicated by depression has become an imperative priority in clinical practice.

In recent years, immune checkpoint inhibitors represented by PD-1 monoclonal antibodies have been applied in the treatment of various malignant tumors, yet these agents may induce immune system attacks on normal tissues, leading to multi-organ immune-related adverse events, including those involving the central nervous system (3, 4). Meanwhile, fluoxetine, a well-recognized and widely used selective serotonin reuptake inhibitor (SSRI) for depression, has been reported to correlate with reduced cancer incidence and mortality (5, 6). However, it remains unclear whether these two agents exert synergistic effects in comorbid hepatocellular carcinoma and depression, warranting further validation and elucidation of their potential regulatory genes and networks.

Accumulating studies have confirmed that aberrant tryptophan metabolism and subsequent immune dysregulation serve as the core pathological crosstalk driving the co-occurrence of hepatocellular carcinoma (HCC) and depression (7, 8).Tryptophan metabolic disturbance is a classic immunometabolic disorder that triggers abnormal immune cell infiltration and breaks immune homeostasis, which is closely involved in the progression of both tumors and neuropsychiatric diseases. Based on this immunometabolic background, we hypothesized that key candidate genes mediate HCC-depression comorbidity by modulating tryptophan-dependent immune disorders. Through integrated multi-omics bioinformatic analysis and further *in vivo* validation, we finally screened out three core candidate genes related to comorbid immune dysregulation: *BTN3A3*, *GLCE*, and *RBCK1*. In the immune regulatory pathway, Published studies indicated that *BTN3A3* participates in the activation of immune cells such as γδT cells (9, 10); *GLCE* is involved in immune cell recruitment and localization (11, 12); *RBCK1* contributes to the implementation of immune-related functions (13, 14). Of note, *BTN3A3* is a primate-specific gene without functional rodent orthologs, limiting its *in-vivo* functional validation in mouse models. To address these gaps, the present study integrated multi-omics bioinformatics, *in vivo* animal tests, multi-omics profiling and molecular docking to dissect core genes and immunometabolic mechanisms of HCC-depression comorbidity, and further explore viable combination therapeutic regimens.In the signal transduction pathway, they collectively regulate inflammation-related pathways such as the NF-κB pathway (15, 16). In the cellular homeostasis pathway, *GLCE* and *RBCK1* act synergistically to preserve the stability of the cellular microenvironment (17, 18). Among them, *GLCE* shows a more prominent correlation with tryptophan-related immune dysregulation in comorbid states.

This study aimed to dissect the molecular mechanisms underlying hepatocellular carcinoma (HCC)-depression comorbidity and identify potential therapeutic targets related to tryptophan-driven immunometabolism. Of note, *BTN3A3* is a primate-specific gene and lacks functional orthologs in rodents, which prevents its direct functional verification in mouse models. To fill the above research gaps, we integrated multi-omics bioinformatic mining, *in-vivo* animal experiments, targeted metabolomics detection and molecular docking simulation to explore core genes and immunometabolic mechanisms for HCC-depression comorbidity and evaluate the synergistic therapeutic potential of fluoxetine combined with anti-PD-1.

## 2 Materials and methods

### 2.1 Animal grouping, model establishment, and drug administration

Eight-week-old male C57BL/6 mice (20–25g) were obtained from Beisifu Experimental Animal Technology Co., Ltd. (Beijing, China, 2025–0166). All mice were maintained under standardized housing conditions with ad libitum food and water access, a 12-h light/dark cycle (lights on at 08:00), ambient temperature of 22 ± 2 °C and relative humidity 50%–60%. All experimental protocols were approved by Shanxi University of Traditional Chinese Medicine (approval number: AWE202503200). SPF-grade healthy male C57BL/6J mice were randomly divided into five groups (n=12): Control, Tumor+CUMS, fluoxetine (FXT, 5 mg·kg^-1^), anti-PD-1 (5 mg·kg^-1^), and anti-PD-1+FXT. Except the Control group, all other mice were simultaneously established with subcutaneous Hepa1-6 hepatoma xenograft and chronic unpredictable mild stress (CUMS) models. Four animals were excluded during the experiment due to death or excessive tumor burden, leaving n=8 per group for *in vivo* phenotypic measurements, and subsets of n=3 per group were selected for subsequent molecular detection assays. Anti-PD-1 antibody was intraperitoneally injected every 5 days for the anti-PD-1 group, while the combination group received both agents synchronously. Body weight was monitored throughout the experiment. All non-Control mice were subjected to CUMS stimulation. After final administration, mice were fasted overnight, anesthetized and sacrificed for sample collection. Serum, tumor, liver, small intestine and brain tissues were isolated, snap-frozen in liquid nitrogen and stored at −80 °C. Detailed CUMS stressors and weekly random schedules are provided in **Supplementary Tables S1**, **S2**.

### 2.2 Behavioral tests

All behavioral tests were conducted uniformly in a quiet room specifically designed for behavioral assays, with the researchers blinded to treatment allocation. A 10-minute habituation phase was allowed prior to each test, including the Sucrose Preference Test (SPT), Forced Swimming Test (FST), Three-Box Social Interaction Test (TBSIT), and Tail Suspension Test (TST). Detailed experimental protocols are provided in the references (19).

### 2.3 Hematoxylin and eosin staining

Paraffin-embedded tumor tissues were sectioned at 5 μm thickness. The sections were dewaxed twice in xylene for 20 min and rehydrated in anhydrous ethanol twice for 5 min, followed by treatment with 75% ethanol and water. The sections were stained with hematoxylin at room temperature for 3–5 min, then dehydrated in 85% and 95% ethanol before eosin staining. After dehydration in anhydrous ethanol and clearing in xylene, the sections were sealed with neutral gum. Cell morphology was observed, and images were captured under a light microscope at 400× magnification.

### 2.4 Western blotting

Total cellular proteins were extracted from mouse prefrontal cortex (PFC), tumor, and liver tissue samples using a Total Protein Extraction Reagent Kit (KeyGEN). Thirty μg of protein was separated by 10% SDS-PAGE and transferred onto PVDF membranes via wet-electrotransfer. Membranes were blocked with 5% non-fat milk for 2 h at room temperature. After thorough washing with TBST, membranes were incubated overnight at 4 °C with primary antibodies against GLCE (1:1000, Proteintech, 33606-1-AP), RBCK1 (1:1000, Abcam, ab309104), and GAPDH (1:5000, Affinity Biosciences, AF7021). Following further TBST washes, membranes were incubated with horseradish peroxidase-conjugated goat anti-rabbit IgG secondary antibody (1:5000, Affinity Biosciences, S0001). Protein bands were visualized using an enhanced chemiluminescence kit (Epizyme) and a Molecular Imager system (Bio-Rad). Band intensity was quantified by Image-J software for semi-quantitative analysis.

### 2.5 Immunohistochemistry

Mouse prefrontal cortex (PFC) and tumor tissues were cut into 3-4 μm-thick serial sections for immunohistochemical staining. Following antigen retrieval according to the manufacturer’s instructions, sections were incubated overnight at 4 °C with primary antibodies against Ki-67 (1:1000, Cell Signaling Technology, #12202T) and GLCE (1:1000, Proteintech, 33606-1-AP), followed by incubation with horseradish peroxidase-conjugated secondary antibody (1:500, Abcam) for 2 h. After three washes with PBS (8 min per wash), sections were visualized using DAB chromogenic reagent, counterstained with hematoxylin, dehydrated, cleared, and mounted with neutral gum. Images were captured under 200× magnification. Negative controls omitting primary antibodies were included in each staining batch.

### 2.6 TCGA database (https://portal.gdc.cancer.gov/)

We downloaded liver cancer mRNA expression profiles from the TCGA database, yielding a total of 424 samples, including 50 normal and 374 tumor specimens (20, 21). Raw read count data were normalized, followed by log_2_(*x* + 1) transformation to stabilize expression variance. Probe-level signals were aggregated to gene-level expression values for downstream analyses.

### 2.7 GEO database (https://www.ncbi.nlm.nih.gov/geo/info/datasets.html)

All datasets were retrieved from the NCBI GEO database. GSE80655 (platform GPL11154) contained Series Matrix files with transcriptomic profiles of 139 samples (70 controls and 69 major depressive disorder (MDD) patients). Single-cell transcriptomic datasets included GSE213982 (72 samples) and GSE166635. Spatial transcriptomic data were obtained from GSE245908, covering 2 liver cancer samples. For downloaded GEO expression matrices, multiple probes mapped to the same gene were merged by taking the maximum expression value when applicable, and log_2_(*x* + 1) transformation was applied prior to statistical testing.

### 2.8 Exposure data

eQTL data was obtained from the eQTLGen consortium (22) (*https://www.eqtlgen.org*) database. The eQTLGen consortium has entered its second phase, conducting large-scale blood-based genome-wide meta-analyses (23).

### 2.9 Outcome data

Participants in the liver cancer and depression GWAS were predominantly of European ancestry (24). Outcome summary statistics were sourced from the FinnGen biobank. Within FinnGen, liver cancer and depression cases were identified using corresponding International Statistical Classification of Diseases (ICD) codes. Liver cancer included 947 cases and 378,749 controls; depression included 59,333 cases and 434,831 controls.

### 2.10 Statistical analysis

All statistical analyses were performed using *R* software (v4.3.0). For *in vivo* animal experiments, data normality and homogeneity of variance were assessed prior to statistical testing. Comparisons between two groups were conducted using unpaired Student’s t-test, while one-way analysis of variance (ANOVA) followed by Tukey’s post-hoc test was applied for multiple-group comparisons. Correlation analysis was performed using *Pearson* correlation. For multi-omics bioinformatic analyses, MR (25, 26), colocalization, GSVA, GSEA, PCA, OPLS-DA and enrichment analyses were implemented as described above. *P<*0.05 was considered statistically significant. Detailed parameters and packages used for bioinformatic statistics are summarized in **Supplementary Tables S3–S5**.

### 2.11 Sensitivity analysis

We performed MR leave-one-out sensitivity analysis to evaluate the influence of individual genetic variants on liver cancer and depression risk (27). This plot presents effect estimates generated by sequentially omitting each SNP, together with the overall estimate derived from all variants. Comparison of these values identifies whether exclusion of individual SNPs changes the pooled result, thus assessing analytical robustness.

### 2.12 Heterogeneity test

We calculated the weighted sum of squares based on the effect size and standard error of each SNP to obtain the Q statistic (28, 29) The Q statistic follows a chi-square distribution with degrees of freedom equal to the number of SNPs minus one. A *P*-value > 0.05 for the Q statistic indicates no significant heterogeneity among SNP effect sizes, suggesting consistent genetic effects on disease risk.

### 2.13 Colocalization analysis

We performed colocalization analysis using the coloc method and eQTL summary data and GWAS of liver cancer/depression (30, 31). Posterior probabilities were calculated within a 100-kb window flanking each index SNP. In coloc outputs, H3 denotes the posterior probability of association driven by distinct causal variants for gene expression and disease, whereas H4 corresponds to shared causal variants. We set SNP.PP.H4 > 0.90 as the threshold for significant colocalization.

### 2.14 GSEA&GSVA analysis

Based on the expression of key genes, samples were divided into high and low expression groups. GSEA (32–34) was performed to identify intergroup signaling pathway differences, using version 7.0 annotated gene sets from the MsigDB database as the background. Enriched gene sets with adjusted P < 0.05 were ranked by normalized enrichment score. Gene set variation analysis (GSVA), a nonparametric unsupervised approach (35–37), was further used to compute pathway enrichment scores and evaluate inter-sample variation in biological function.

### 2.15 Immune infiltration

CIBERSORT is a widely-used algorithm for quantifying immune cell composition within tissue microenvironments (38, 39). It employs 547 signature genes from the LM22 matrix to deconvolve 22 human immune cell phenotypes, such as T cells, B cells, plasma cells, and myeloid subsets. We applied the CIBERSORT algorithm to estimate the relative fractions of these 22 immune infiltrates and examine correlations between gene expression and immune cell abundance.

### 2.16 Transcription factor regulatory network

This study predicts transcription factors by using the R package “RcisTarget”. All calculations performed by RcisTarget are based on motifs (40, 41). A motif’s normalized enrichment score (NES) relies on the total number of motifs present in the database. Beyond annotations provided by raw data, additional annotations were inferred from motif similarity and gene sequences (42). To assess motif overrepresentation within gene sets, we first computed the AUC for each motif-set pair derived from gene-set recovery ranking curves. Each motif’s NES was then determined according to the AUC distribution of all motifs in the corresponding gene set.

### 2.17 Non-coding RNA network associated with key genes

We further explored whether miRNAs targeting key genes modulate transcription or degradation of risk-related genes (43, 44). We retrieved miRNAs associated with key genes from the miRcode database and visualized the gene-miRNA network using Cytoscape.

### 2.18 Quality control

Expression matrices were imported using the Seurat package. Cells were filtered according to UMI counts, detected gene numbers, as well as mitochondrial and ribosomal read fractions per cell (45). Outliers were defined as values exceeding three median absolute deviations (MAD) from the median for nCount_RNA, nFeature_RNA or percent.mt. Cells with excessively high UMI and gene counts were considered doublets, and cells with elevated mitochondrial and ribosomal read fractions were regarded as low-quality cells prone to apoptosis or cell debris (46). After the above filtering steps, DoubletFinder (v2.0.4) was further applied to remove doublets. After QC, 15,921 cells (HCC dataset) and 126,555 cells (depression dataset) were retained for downstream analysis.

### 2.19 Data standardization

Data were log-normalized using the NormalizeData function (47), followed by cell cycle scoring via CellCycleScoring (48) and highly variable genes were identified by FindVariableFeatures. A total of 2,000 highly variable genes were identified using the vst method in FindVariableFeatures. ScaleData was applied to regress out confounding effects derived from mitochondrial genes, ribosomal genes and cell cycle variation. Principal component analysis was performed with 50 principal components via RunPCA, followed by Harmony batch correction (grouped by sample) and UMAP embedding using dimensions 1–20. Clustering was evaluated across a resolution grid ranging from 0.2 to 1.2, and a final resolution of 0.2 was selected, yielding 12 clusters (HCC) and 17 clusters (depression). Clusters were annotated using the CellMarker database and canonical marker genes from literature, with auxiliary annotation by SingleR, resulting in 9 and 7 annotated cell types for HCC and depression datasets, respectively.

### 2.20 RCTD deconvolution

We leveraged annotated scRNA-seq data to define cell type-specific profiles of cell types expected to be present in spatial transcriptomics data (49, 50). Supervised cell type classification faces the challenge of platform effects. Studies indicate that supervised approaches often fail without accounting for such effects, as systematic technical variation overwhelms biological signals. These biases have been observed in matched single-cell and single-nucleus RNA-seq (snRNA-seq) datasets. RCTD mitigates platform effects and enables spatial transcriptome deconvolution across different platforms. It accurately identifies cell-type localization in both simulated and empirical spatial transcriptomic data.

### 2.21 Spatial cell interactions

MISTy facilitates the interpretation of marker interactions by modeling intra- and intercellular relationships. It constructs models to characterize distinct spatial contexts, representing regulatory patterns including intracellular and paracrine signaling (51, 52). MISTy constructs a separate model component termed a view for each interaction type. Each view represents a potential source of variation in measured marker expression (53). The contribution of each view to individual marker expression is subsequently quantified. These estimates reflect interactions originating from distinct spatial contexts and are derived from view-specific models.

### 2.22 Molecular docking

The specific steps of molecular docking were referred to the literature (19).

### 2.23 Flow cytometry

Whole-blood samples were collected from mice at the experimental endpoint. Peripheral blood mononuclear cells (PBMCs) were isolated using lymphocyte separation medium via standard density-gradient centrifugation protocols. Isolated PBMCs were washed twice with pre-cooled phosphate-buffered saline (PBS). After adding the staining buffer, the cells were vortexed, centrifuged, and the supernatant was discarded; this washing step was repeated once. Cells were incubated with anti-CD206 (1:2000, BioLegend, 321106). The labeled cells were then analyzed on FASCanto II (Becton Dickinson, USA), and data were processed using FlowJo X 10.0.7 software (Becton Dickinson, USA).

### 2.24 Targeted UHPLC-MS/MS analysis of tryptophan metabolites

Serum and tissue samples were collected from identical mice used for PBMC isolation. We performed UHPLC-MS/MS to absolutely quantify 38 tryptophan pathway metabolites following Personalbio’s validated protocol, with stable isotope internal standards added during cold methanol extraction. Metabolites were separated on a Waters BEH C18 column via gradient elution and detected on a triple quadrupole mass spectrometer in MRM mode. Calibration curves were built for absolute quantification. Pooled QC samples were interspersed throughout runs; all metabolites had CV<0.3, indicating stable detection. Metabolite data were UV-scaled, then analyzed by PCA, PLS-DA and OPLS-DA with permutation tests to prevent overfitting. Differential metabolites were screened by Student’s t-test (*P* < 0.05) and OPLS-DA VIP > 1, followed by KEGG enrichment via clusterProfiler to characterize disturbed tryptophan metabolism.

### 2.25 Enzyme-linked immunosorbent assay

Mouse serum samples were obtained from the same peripheral blood specimens used for PBMC isolation. Commercial mouse IL-10 (Cat. No. E-EL-M0046) and TGF-β (Cat. No. E-EL-M0051) ELISA kits were purchased from Elabscience (Wuhan, China). Serum IL-10 and TGF-β levels were quantified strictly following the manufacturer’s instructions. Optical density values were read with a microplate reader, and sample concentrations were calculated against standard curves.

### 2.26 Integrated transcriptomic and targeted tryptophan metabolomic analysis

RNA-seq transcriptomic data and UHPLC-MS/MS targeted metabolomic data of mouse serum were combined to explore the regulatory landscape of tryptophan metabolism. Pearson correlation analysis was performed to calculate the correlation coefficients between differentially expressed genes (DEGs) and differential tryptophan-related metabolites, and gene-metabolite correlation networks were established based on significant correlation pairs. Joint KEGG enrichment analysis of DEGs and differential metabolites was conducted to screen co-enriched tryptophan metabolic pathways, which integrated transcriptional alterations and metabolic phenotypes to clarify the molecular mechanism underlying the disease model.

## 3 Results

### 3.1 Interventional effects of fluoxetine combined with anti-PD-1 in HCC-CUMS comorbid mice

We established a mouse comorbidity model of hepatocellular carcinoma (HCC) complicated with depression via subcutaneous Hepa1-6 tumor inoculation combined with chronic unpredictable mild stress (CUMS), and treated model mice with fluoxetine (FXT), anti-PD-1 monotherapy, and their combination.(**Figure 1A**). Body weight was dynamically recorded throughout administration (**Figure 1B**). Upon sacrifice, tumor tissues were dissected and measured. Single administration of FXT or anti-PD-1 moderately suppressed tumor progression, and the combined regimen achieved the strongest inhibitory effect on tumor volume and weight. (**Figures 1C, D**). Histological H&E staining (400×) revealed alleviated inflammatory infiltration within tumor lesions, and Ki-67 immunohistochemistry (400×) showed suppressed tumor cell proliferation in all three treatment groups compared with the untreated Tumor+CUMS model group (**Figure 1E**). For neurobehavioral assessment: the comorbid model exhibited obvious depressive-like behaviors shown by declined sucrose preference (SPT, **Figure 1F**) and prolonged immobility duration in forced swim test (FST, **Figure 1G**). Fluoxetine single treatment markedly alleviated depressive behaviors, while anti-PD-1 alone produced negligible antidepressant efficacy. The combination of fluoxetine and anti-PD-1 exerted prominent rescue effects on depression-like symptoms. Heatmaps from the three-chamber social interaction test (**Figure 1H**) further confirmed that only combined intervention effectively reversed social avoidance of comorbid mice.

**Figure 1:**
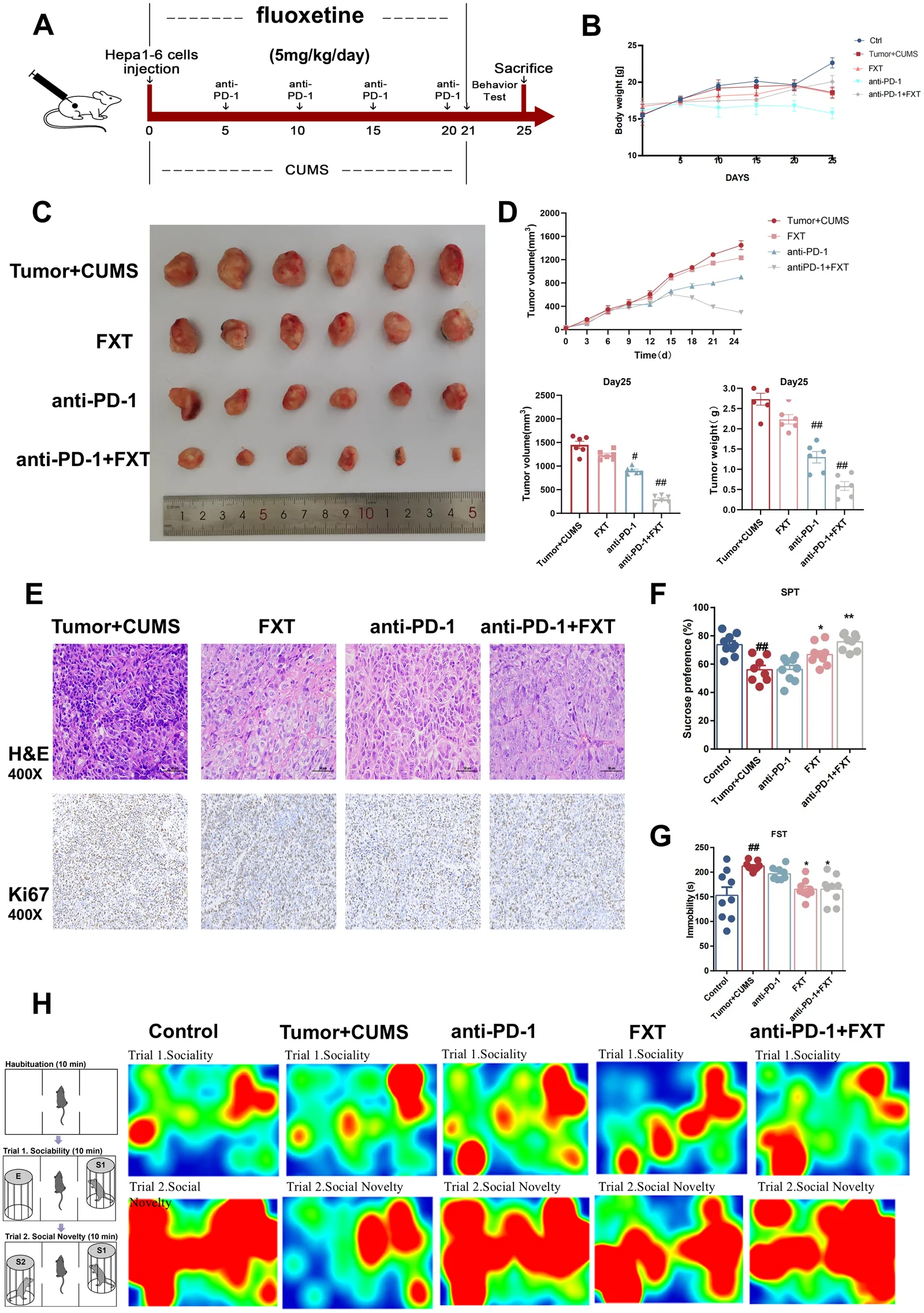
Therapeutic effects of fluoxetine combined with anti-PD-1 in HCC-CUMS comorbid mice. **(A)** Schematic timeline for subcutaneous Hepa1-6 tumor inoculation, chronic unpredictable mild stress (CUMS) modeling, and drug administration. **(B)** Dynamic body weight curves of mice during the experimental period. **(C,D)** Representative tumor morphology, tumor volume and tumor weight statistics. **(E)** H&E staining and Ki-67 IHC staining of tumor tissues (400× magnification). **(F,G)** Sucrose preference test (SPT) and forced swimming test (FST) for depression-like behaviors. **(H)** Heatmap of three-chamber social interaction test. Compared with the blank group, ^#^*p* < 0.05 or ^##^*p* < 0.01; compared with the model group, ^*^p < 0.05 or ^**^p < 0.01. n=8.

### 3.2 MR and colocalization analyses identify seven shared genes linking hepatocellular carcinoma and depression

To explore the shared genetic basis of hepatocellular carcinoma (HCC) and depression, we conducted two-sample Mendelian randomization (MR) combined with GWAS-eQTL colocalization analyses. Manhattan and volcano plots displayed genome-wide candidate risk loci for HCC and depression, respectively (**Figures 2A, B**). Under the IVW threshold of *P* < 0.05, preliminary MR screening retained 374 candidate signals for HCC from 458 initial MR hits and 656 signals for depression from 885 candidates. We further filtered 223 positive causal signals for HCC and 350 for subsequent analysis (**Figure 2C**). Venn diagram of positive MR outcomes identified seven overlapping genes shared by HCC and depression, including two protective genes (*VPS33B*, *GLCE*) and five risk genes (*RBCK1*, *RAC1*, *FPGT*, *DRD4*, *BTN3A3*) (**Figure 2D**). Scatter plots based on IVW, MR-Egger, weighted-median and weighted-mode methods demonstrated consistent causal effect directions of these seven hub genes on HCC (**Figure 2E**) and depression (**Figure 2F**), meanwhile heterogeneity indexes from MR-Egger and inverse variance weighted calculations further ruled out remarkable heterogeneity and horizontal pleiotropy to consolidate the robustness of causal associations (**Supplementary Figure S1**), and we further predicted binding miRNAs of three pivotal targets (*GLCE*, *RBCK1*, *BTN3A3*) via miRcode database and constructed a Cytoscape-visualized regulatory network covering 67 miRNAs and 110 mRN-miRNA pairs to explore their upstream regulatory patterns (**Supplementary Figure S2**). Taken together, MR and colocalization analyses highlighted seven shared causal genes that may serve as core genetic mediators underlying HCC-depression comorbidity.

**Figure 2:**
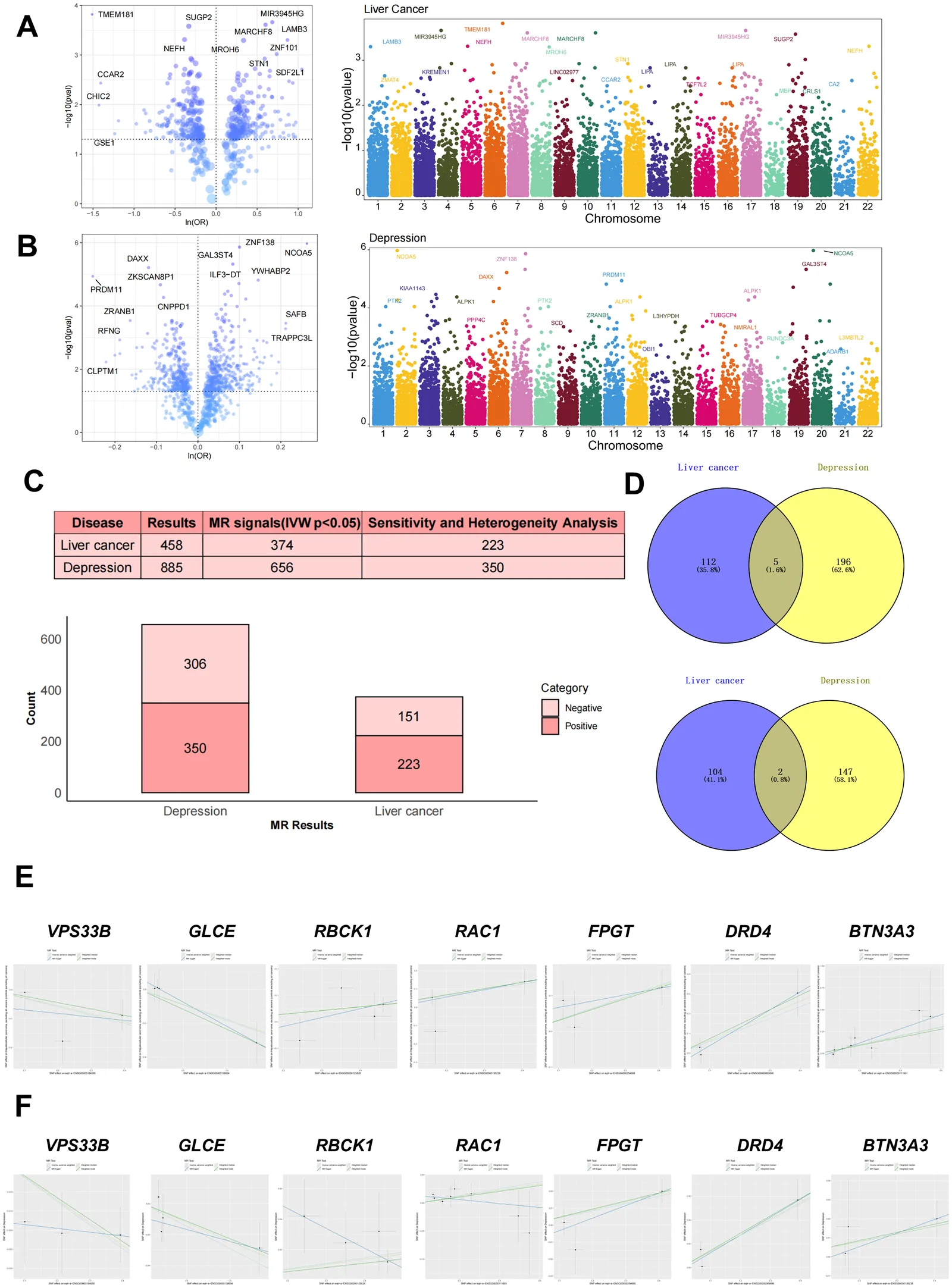
Two-sample eQTL-Mendelian randomization (MR) analysis to identify shared causal genes underlying hepatocellular carcinoma (HCC) and depression. **(A, B)** Volcano plots and Manhattan plots illustrating MR association signals for HCC and depression. **(C)** Summary of MR signals, sensitivity, and heterogeneity analyses, bar charts showing counts of positive MR hits for each disease. **(D)** Venn diagrams displaying overlapping causal genes between HCC and depression. **(E, F)** MR scatter plots of seven hub genes for HCC and depression separately.

### 3.3 Genetic colocalization features of hub genes *GLCE*, *RBCK1* and *BTN3A3* across HCC and depression loci

We next integrated GWAS and eQTL datasets to conduct genetic colocalization for the seven shared candidate genes. Significant colocalization signals were verified for *GLCE*, *RBCK1* and *BTN3A3* in both HCC and depression, with all *SNP.PP.H4* > 0.90 (**Figure 3A**). LocusZoom plots visualized regional colocalization patterns in liver and depression-relevant brain tissues separately (**Figures 3B, C****).** *RBCK1* displayed the sharpest colocalization peak with compact LD block, and high-LD variants (*r²*>0.8) tightly clustered around the top signal without scattered noise; *GLCE* yielded robust overlapping peaks in both phenotypes with concentrated high-LD signals at the summit. Although *BTN3A3* displayed a wider LD range and minor secondary peaks, its dominant peak still covered risk loci shared by the two diseases, supporting reliable colocalization. These colocalization results implied that disease-associated variants likely regulate disease susceptibility by modulating gene transcription instead of altering protein-coding sequences. Among the three colocalized genes, *GLCE* and *RBCK1* were prioritized as primary candidate targets for subsequent *in vivo* validation, while *BTN3A3* was only analyzed via public multi-omics data due to its absence in mice. Transcription factor enrichment analysis targeting the three hub genes identified multiple common cis-regulatory motifs. The motif cisbp_M2272 bound by E2F4 ranked highest with an NES of 7.38 (**Figure 3D**).

**Figure 3:**
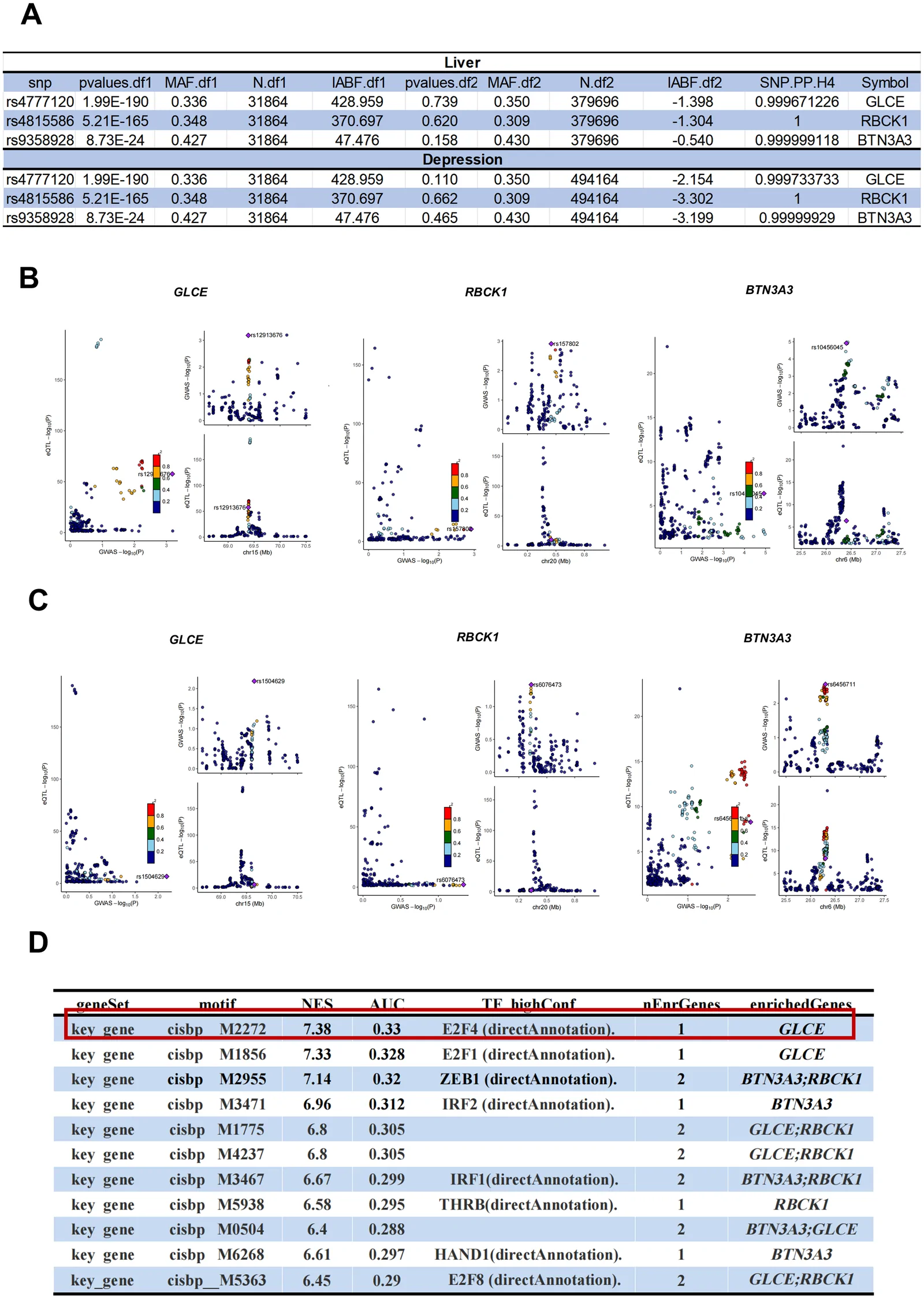
Genetic colocalization and transcriptional regulation analysis of *GLCE*, *RBCK1* and *BTN3A3* **(A)** Summary statistics of eQTL-GWAS colocalization signals in liver and brain tissues for the three hub genes. **(B, C)** LocusZoom colocalization plots of hub genes in HCC and depression-related genomic regions. **(D)** Transcription factor enrichment table highlighting top transcription factors targeting GLCE, RBCK1 and BTN3A3. The red box indicates the transcription factor with the highest NES score.

### 3.4 GSVA and GSEA reveal shared inflammatory pathways correlated with altered tryptophan metabolism in HCC-depression comorbidity

We performed gene set variation analysis (GSVA) and gene set enrichment analysis (GSEA) to characterize functional pathways associated with *GLCE*, *RBCK1* and *BTN3A3* in both hepatocellular carcinoma and depression datasets. GSVA distribution curves and circular regulatory networks revealed that all three hub genes were closely associated with disordered tryptophan-related immunometabolic pathways during HCC development (**Figure 4A**). In HCC samples, *GLCE* was enriched in pro-inflammatory and tumor proliferative pathways; *RBCK1* participated in immune regulation and tryptophan metabolic processes; *BTN3A3* correlated with immune activation signaling that facilitates tumor progression. For depression, *GLCE* disturbed neuroimmune homeostasis via inflammatory metabolic signaling; *RBCK1* modulated systemic immune-inflammatory balance; *BTN3A3* regulated synaptic function and immune pathways to induce abnormal neuronal activity (**Figure 4B**).

**Figure 4:**
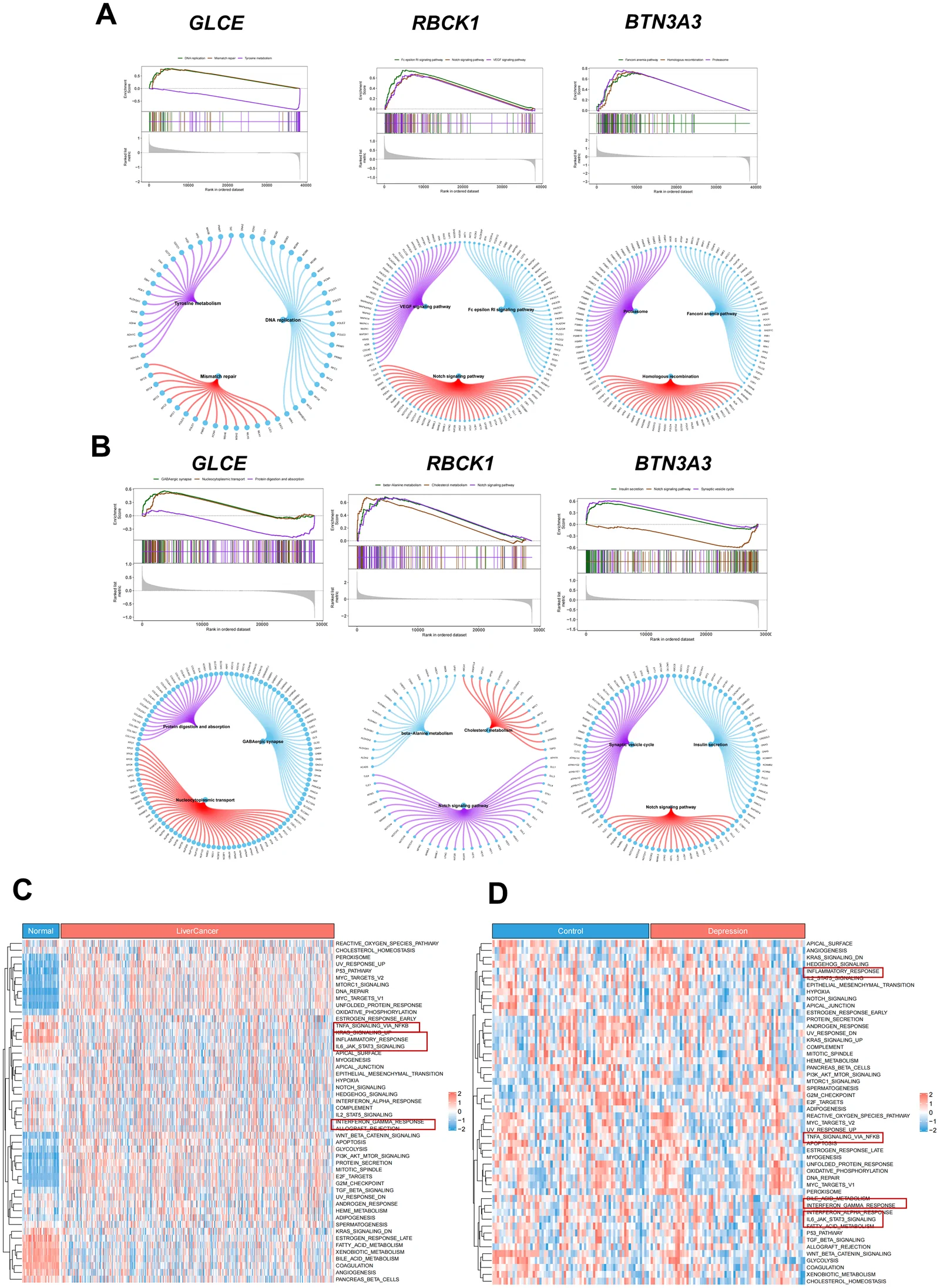
Gene set variation analysis (GSVA) and gene set enrichment analysis (GSEA) of *GLCE*, *RBCK1* and *BTN3A3* in HCC and depression transcriptomic datasets. **(A, B)** GSVA pathway activity distribution curves and circular regulatory networks for each gene in HCC and depression. **(C, D)** GSEA enrichment heatmaps showing significantly perturbed biological pathways correlated with hub gene expression.

Cohort-level GSEA corroborated these findings (**Figures 4C, D**). In HCC transcriptomes, significantly enriched pathways covered TNFA/NF-κB signaling, inflammatory response, IL6-JAK-STAT3 axis and interferon gamma response, all key inflammatory cascades regulated by tryptophan-kynurenine metabolites. Similar immune and inflammatory signatures were also enriched in depression datasets. Collectively, these data implied that disturbed tryptophan metabolism may induce shared inflammatory signaling alterations to drive HCC-depression comorbidity.

### 3.5 Immune infiltration landscape and correlations of hub genes with M2 macrophage-mediated immunosuppression

We further explored immune cell infiltration profiles and the correlation between *GLCE*, *RBCK1*, *BTN3A3* and immunosuppressive microenvironment in HCC and depression cohorts. Immune cell infiltration boxplots indicated markedly elevated proportions of M2-type macrophages in both tumor and depression samples compared with corresponding control groups (**Figure 5A**). Flow cytometry results of peripheral immune cells from mouse models showed that the proportion of CD206^+^M2 macrophages was significantly reduced in the fluoxetine combined anti-PD-1 treatment group relative to the Tumor+CUMS comorbidity model group (**Figure 5B**). Correlation heatmaps demonstrated that the expression levels of the three hub genes were positively correlated with M2 macrophage abundance and multiple immunosuppressive immune subsets (**Figure 5C**). ELISA quantification of serum immunosuppressive cytokines revealed that IL-10 and TGF-β concentrations were remarkably increased in Tumor+CUMS mice; single fluoxetine or anti-PD-1 treatment partially lowered cytokine levels, while combined intervention produced the most obvious inhibitory effect on IL-10 and TGF-β secretion (**Figure 5D**). Collectively, these data suggested that *GLCE*, *RBCK1* and *BTN3A3* might participate in M2 macrophage polarization and subsequent immunosuppressive cytokine release under HCC-depression comorbid status.

**Figure 5:**
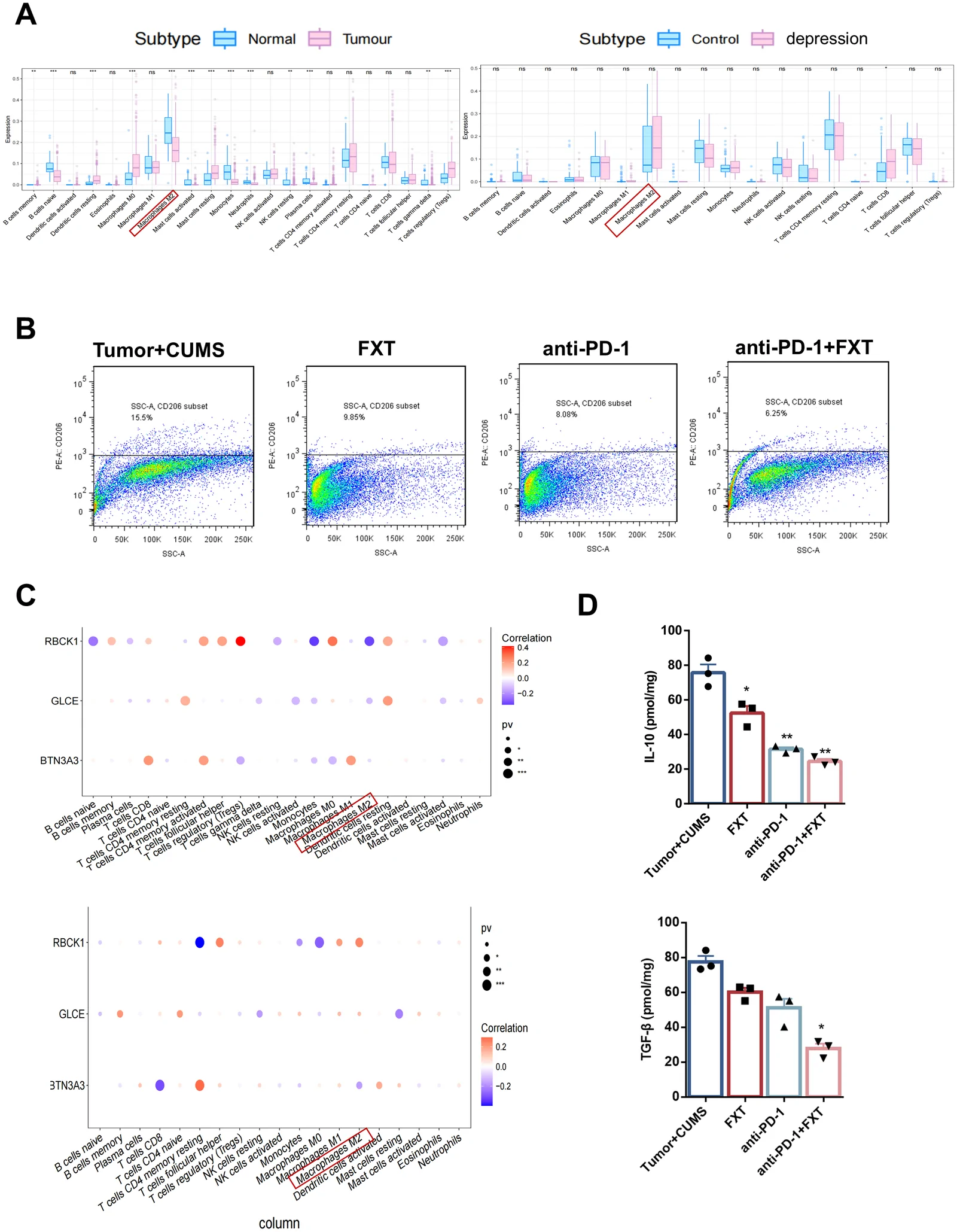
Immune cell infiltration landscape and correlation between hub genes and M2 macrophage-mediated immunosuppression in HCC-depression comorbidity. **(A)** Box plots of immune cell infiltration proportions comparing normal vs tumor, control vs depression cohorts. **(B)** Representative flow cytometry dot plots for peripheral CD206^+^M2 macrophages from each treatment group. **(C)** Correlation heatmap of *GLCE/RBCK1/BTN3A3* expression with multiple immune cell subsets. **(D)** ELISA quantification of immunosuppressive cytokines IL-10 and TGF-β in mouse serum compared with the model group, ^*^p < 0.05 or ^**^p < 0.01. n=3.

### 3.6 Single-cell transcriptomic profiling reveals hub gene distribution and kynurenine pathway enrichment in human hepatocellular carcinoma

Single-cell RNA-seq analysis was performed on human HCC tissue to characterize the cell-type-specific expression patterns of *GLCE*, *RBCK1* and *BTN3A3*. Raw sequencing data were processed via the Seurat R package. Low-quality cells with fewer than 200 detected genes or abnormal read counts were excluded, and doublet cells were further removed using DoubletFinder, leaving a total of 15,921 qualified cells for downstream analysis. We identified 2,000 highly variable genes, followed by normalization, scaling, PCA and Harmony batch correction (**Supplementary Figures S3–7**). Based on canonical cell marker genes, we annotated 9 distinct cell subsets, including T cells, macrophages, monocytes, B cells, epithelial cells, hepatocytes, fibroblasts, mast cells and endothelial cells (**Figure 6A**). T cell markers CD2 and CD3D, as well as macrophage markers CD163, CD68, exhibited cell-type-specific expression (**Figure 6B**). Feature UMAP and violin plots demonstrated distinct expression signatures for the three hub genes: *BTN3A3* was predominantly enriched in T cells with weak signals in macrophages; *GLCE* showed high expression in hepatocytes and low abundance in fibroblasts; *RBCK1* was highly expressed in endothelial cells and correlated with angiogenesis and cell adhesion functions, with minimal expression in B cells (**Figures 6C–E**). None of the three hub genes displayed prominent expression in other minor cell populations.

**Figure 6:**
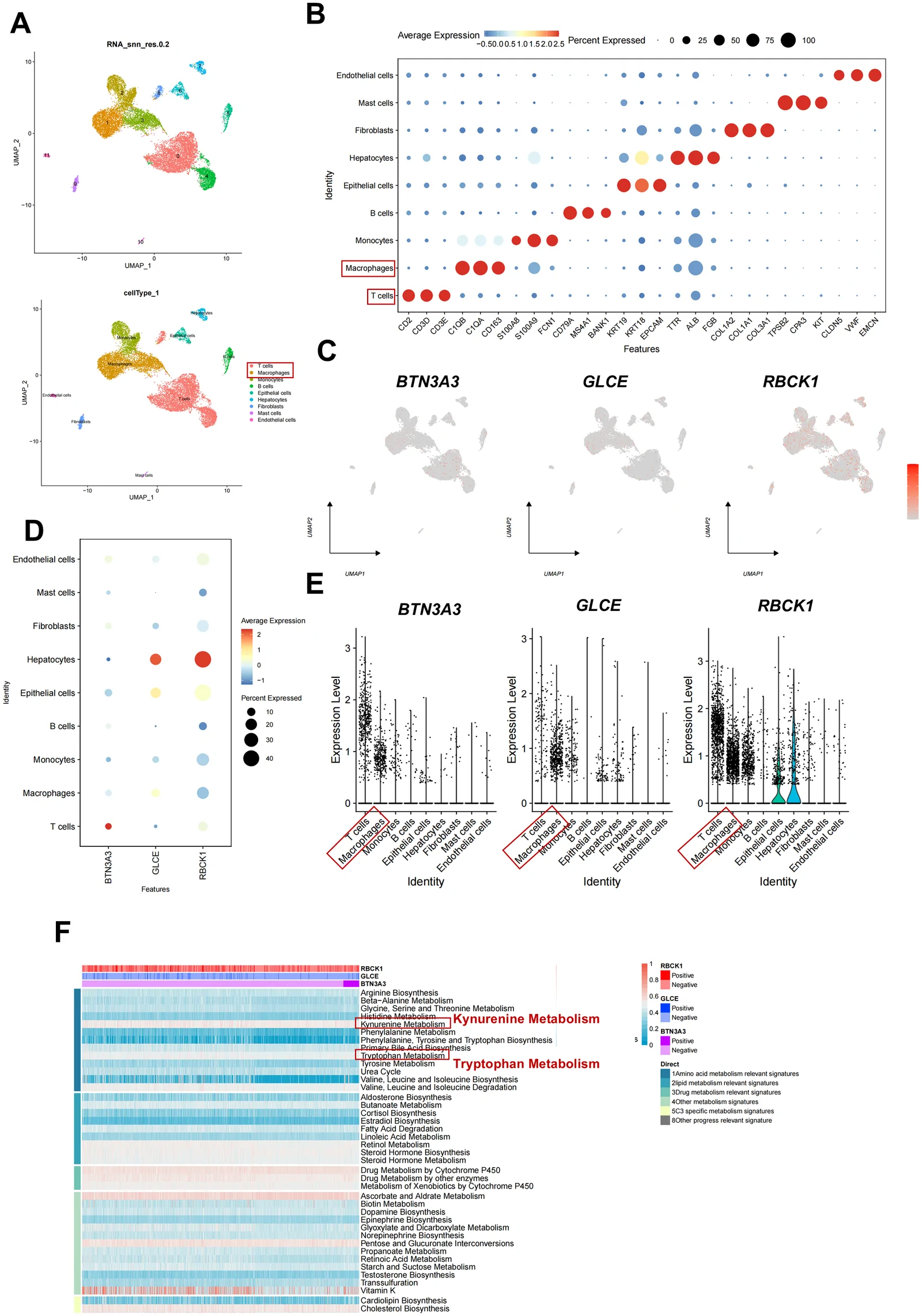
Single-cell transcriptomic profiling of *BTN3A3*, *GLCE* and *RBCK1* in human hepatocellular carcinoma. **(A)** UMAP plots showing cell clustering and cell-type annotation of human HCC single-cell RNA-seq dataset. **(B)** Dot plot of canonical marker genes for tumor-infiltrating cell subset annotation. **(C)** Feature UMAP plots of *BTN3A3*, *GLCE*, and *RBCK1* across tumor single cells. **(D)** Dot plot showing average expression and detection percentage of three hub genes in annotated cell populations. Red boxes indicate T cells and macrophages. **(E)** Violin plots of *BTN3A3*, *GLCE* and *RBCK1* expression across cell types. Red boxes indicate T cells and macrophages. **(F)** Heatmap of pathway-activity signatures correlated with hub-gene expression. Red boxes indicate tryptophan metabolism and kynurenine metabolism pathways. This analysis was performed on public single-cell RNA-seq dataset.

Pathway activity heatmaps indicated prominent enrichment of the kynurenine metabolism pathway, accompanied by elevated expression of its rate-limiting enzyme genes. Additional enriched pathways included cytochrome P450 drug metabolism, ascorbate and aldarate metabolism, and cholesterol biosynthesis (**Figure 6F**). These single-cell transcriptomic data implied that the three hub genes may participate in the modulation of kynurenine metabolic signaling within HCC cell subpopulations.

### 3.7 Single-cell transcriptomic profiling of brain tissue identifies hub gene expression patterns and kynurenine pathway enrichment in depression

Single-cell RNA-seq of depressive brain tissue was conducted following the identical analytical pipeline used for HCC samples (**Supplementary Figures S8–11**). Cell-type annotation revealed that excitatory neurons, inhibitory neurons and astrocytes constituted the dominant cell populations in brain samples from depressive subjects. Cell subsets marked by *SLC17A7*, *GAD1* and *GFAP* each accounted for over 60% of total cells and exhibited high average gene expression. Microglia and oligodendrocytes represented the secondary abundant populations, whereas oligodendrocyte precursor cells were the rarest cell type(**Figures 7A, B**). We observed cell-type-specific expression patterns for the three hub genes. *GLCE* showed relatively high expression within the astrocyte cluster, with higher expression levels compared with other cell subsets; *BTN3A3* exhibited the highest expression signals in the microglia cluster, with a high proportion of microglia cells expressing this gene; *RBCK1* displayed prominent expression in excitatory neurons, where less than 20% of cells showed low expression (**Figures 7C–E**). Consistent with the pathway enrichment results in HCC single cells, the kynurenine metabolism pathway was significantly enriched, alongside other pathways including cytochrome P450-mediated drug metabolism and biotin metabolism (**Figure 7F**).

**Figure 7:**
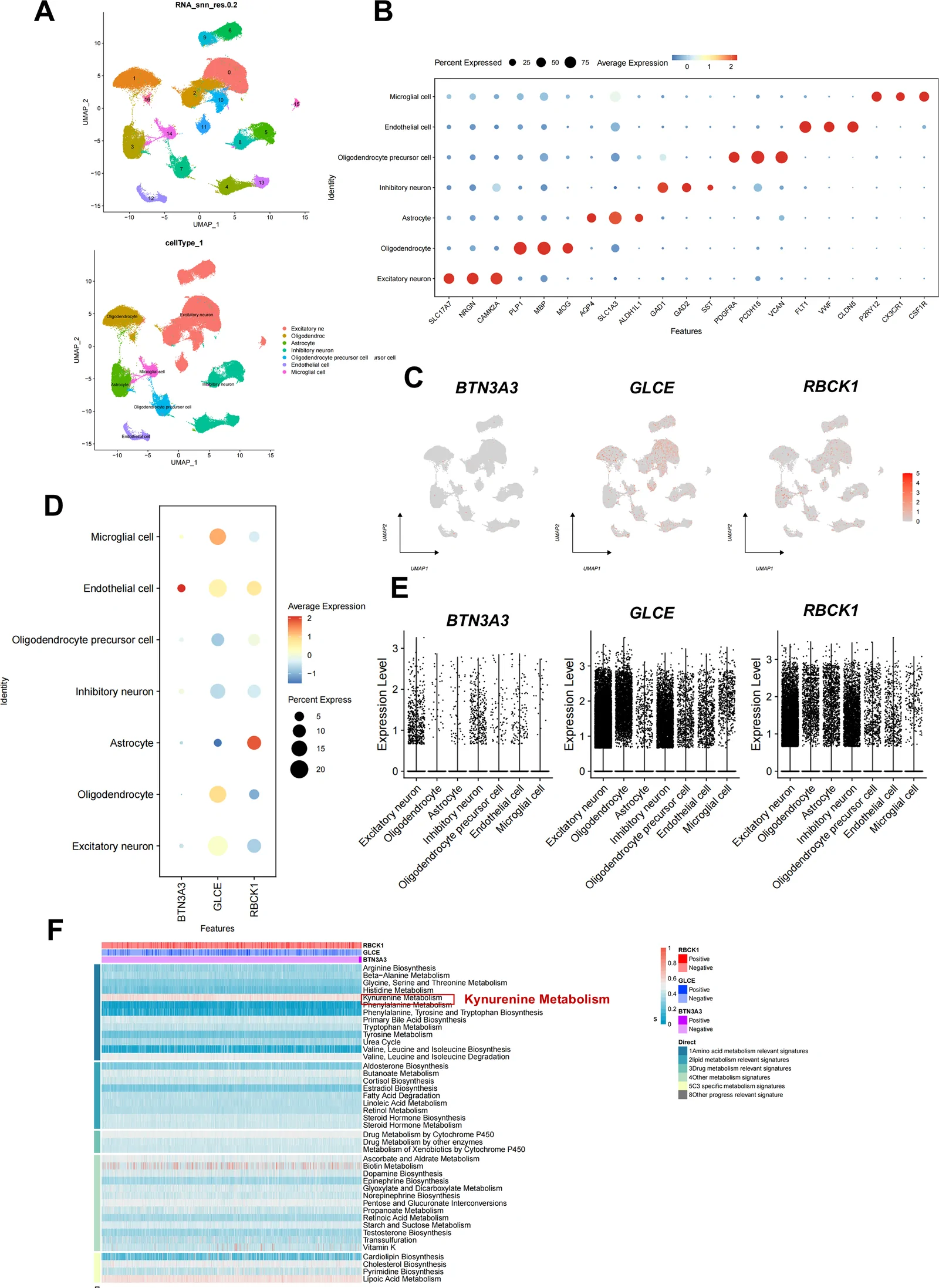
Single-cell transcriptomic profiling of *BTN3A3*, *GLCE* and *RBCK1* in depressive brain tissue. **(A)** UMAP plots showing cell clustering and cell-type annotation of brain single-cell RNA-seq dataset under depressive status. **(B)** Dot plot of canonical marker genes for brain cell subset annotation. **(C)** Feature UMAP plots of *BTN3A3*, *GLCE*, and *RBCK1* across brain single cells. **(D)** Dot plot showing average expression and detection percentage of three hub genes in annotated brain cell populations. **(E)** Violin plots of *BTN3A3*, *GLCE* and *RBCK1* expression across brain cell types. **(F)** Heatmap of pathway-activity signatures correlated with hub-gene expression. Red boxes indicate kynurenine metabolism pathways. All analyses were performed on public single-cell RNA-seq dataset.

### 3.8 Integrated transcriptomic and targeted tryptophan LC-MS/MS metabolomics analysis of brain and serum from HCC-CUMS mice

To systematically characterize transcript-metabolite perturbations linked to tryptophan metabolism under HCC-depression comorbidity, we integrated RNA-seq transcriptome data and targeted UHPLC-MS/MS metabolomic profiles of mouse brain and serum samples. Unsupervised PCA exhibited clear metabolic separation among experimental groups in both brain and serum specimens, reflecting distinct metabolic signatures induced by tumor-combined chronic stress and drug interventions (**Figure 8A**). Targeted quantitative detection demonstrated markedly reduced L-tryptophan (L-Trp) and elevated L-kynurenine (L-Kyn) in the Tumor+CUMS model group; fluoxetine plus anti-PD-1 combination therapy largely restored the abnormal levels of the two core metabolites (**Figure 8B**). Bar chart and box plot exhibited altered L-Trp abundance between Tumor+CUMS (M) and combination (SP) groups (**Figure 8C**). Pairwise PCA score plots further displayed inter-group metabolic divergence for M vs P, M vs SP, and P vs SP comparisons (**Figure 8D**). Volcano plots identified abundant differential metabolites between pairwise groups, with L-tryptophan and 2-Aminophenol annotated as representative altered metabolites (**Figure 8E**). Bidirectional hierarchical clustering heatmaps displayed distinct expression clustering patterns of indole-series differential metabolites across core experimental groups (**Figure 8F**). Joint KEGG enrichment bubble plots integrating transcriptomic differentially expressed genes and LC-MS differential metabolites consistently pinpointed tryptophan metabolism as the most significantly perturbed pathway (mmu00380). The bubble plot further confirmed robust transcriptional and metabolic dysregulation of L-tryptophan-related cascades in the comorbid model, which was partially rescued upon combined fluoxetine and anti-PD-1 intervention (**Figure 8G**). Collectively, multi-omics joint evidence implied that fluoxetine combined with anti-PD-1 could alleviate disordered tryptophan-kynurenine metabolism in HCC-CUMS mice at both transcriptional and metabolic layers.

**Figure 8:**
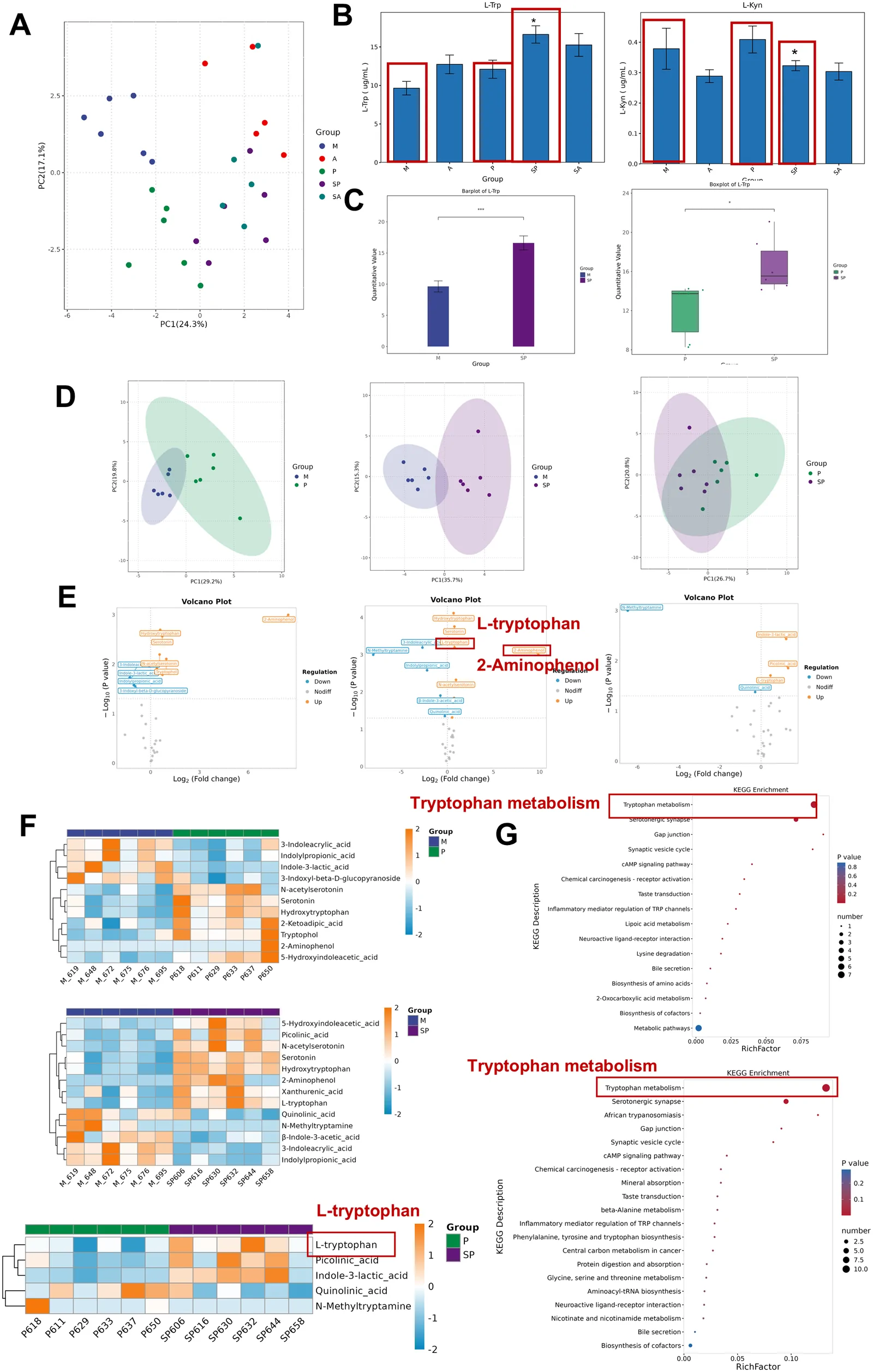
Integrated transcriptome and targeted tryptophan LC-MS/MS metabolomics analysis under combined intervention in HCC-CUMS mice. **(A)** PCA plot showing metabolic separation across brain and serum samples from each experimental group. **(B)** Quantitative bar charts of tissue L-tryptophan (L-Trp) and L-kynurenine (L-Kyn) concentrations measured by targeted LC-MS/MS. **(C)** Bar chart and box plot of L-Trp comparing the Tumor+CUMS (M) group and combination (SP) group. **(D)** PCA score plots showing pairwise metabolic profile comparison between M vs P, M vs SP, and P vs SP groups. **(E)** Volcano plots displaying differential tryptophan-pathway metabolites for pairwise group comparisons. **(F)** Hierarchical clustering heatmaps of indole-series differential metabolites. **(G)** KEGG enrichment bubble plots for the integrated analysis of transcriptomic DEGs and differential metabolites. Red boxes indicate the tryptophan metabolism pathway. Notes: M: Tumor+CUMS comorbidity model group; A: single-agent reference treatment group (not the main focus of this study); P: anti-PD-1 monotherapy group; SP: anti-PD-1+Fluoxetine combination group; SA: alternative agent combined with Fluoxetine reference group.

### 3.9 Spatial transcriptomic multi-omics profiling of human HCC tissues

Spatial transcriptomics combined with multiple bioinformatic pipelines was applied to dissect cell composition, intercellular communication, hub gene spatial distribution and pathway activity within human hepatocellular carcinoma tissues. We analyzed two independent public spatial transcriptomic datasets (GSM7850822, GSM7850823) through RCTD cell-type deconvolution, cell-cell interaction network construction and PROGENy signaling pathway scoring.

RCTD deconvolution was conducted to resolve cell spatial localization across two tissue slices (**Figures 9A, B**). Hepatocytes represented the dominant cell population, whereas epithelial and other stromal cell subsets occupied smaller spatial regions, demonstrating prominent spatial cellular heterogeneity in HCC lesions. Spatial feature plots visualized regional expression of the three hub genes in both tissue specimens (**Figure 9C**). Dot and violin quantification further validated expression differences: *RBCK1* displayed broader, stronger expression signals in GSM7850822 relative to GSM7850823; *GLCE* and *BTN3A3* presented moderate expression restricted to discrete tissue regions, with comparable overall expression abundance between the two samples (**Figures 9D, E**). These findings highlighted inter-sample and intra-tumoral spatial heterogeneity of hub gene transcription.

**Figure 9:**
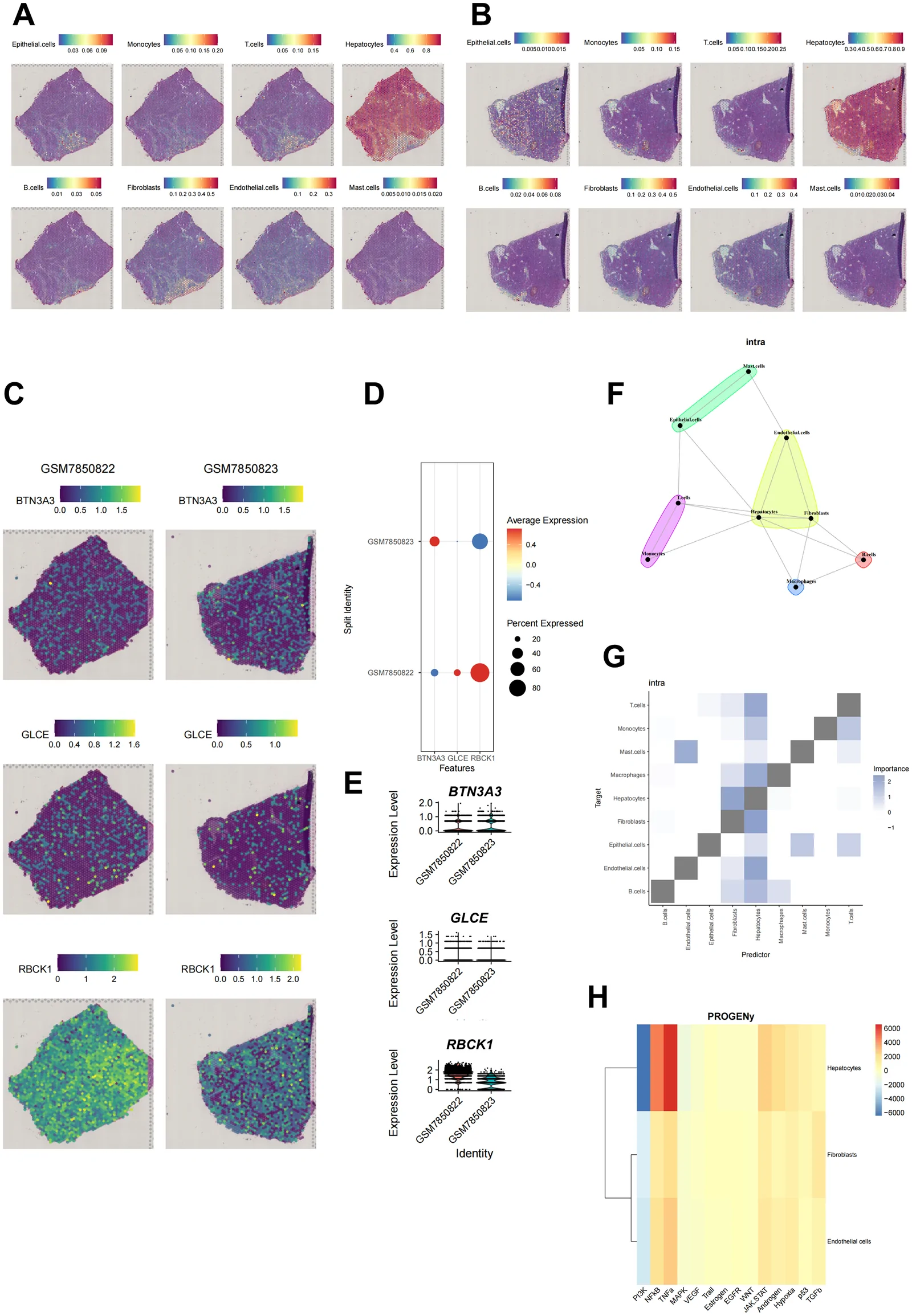
Spatial transcriptomics multi-omics analysis of human HCC tissue datasets (GSM7850822, GSM7850823). **(A, B)** RCTD cell-type deconvolution spatial maps for two independent tissue slices. **(C)** Spatial feature plots showing regional expression of *BTN3A3*, *GLCE* and *RBCK1* in tumor tissue. **(D, E)** Dot and violin plots quantifying hub gene expression levels across spatial transcriptomic samples. **(F)** Cell-cell interaction network diagram of tumor microenvironment cell subsets. **(G)** Heatmap quantifying interaction strength between distinct cell populations. **(H)** PROGENy signaling pathway activity heatmap derived from spatial transcriptome data.

We next constructed a cell-cell communication network and interaction strength heatmap to characterize crosstalk within the tumor microenvironment (**Figures 9F, G**). Hepatocytes exhibited widespread ligand-receptor crosstalk with fibroblasts, endothelial cells and other stromal populations, implying tight regulatory communication across distinct cell compartments in HCC. PROGENy pathway activity scoring revealed cell-type-specific signaling signatures (**Figure 9H**). Collectively, spatial transcriptomic data offered spatial evidence supporting differential signaling regulation among cell subpopulations in HCC tissue.

### 3.10 Binding prediction of fluoxetine with hub proteins and tissue expression verification

We performed molecular docking simulations to predict the binding interaction between fluoxetine and the three hub proteins (**Figure 10A**). Distinct binding conformations and binding-affinity scores were calculated. Lower negative docking energy generally indicates stronger predicted binding capacity. Among the three targets, *BTN3A3* achieved the most favorable docking score, followed by *GLCE* and *RBCK1*. Nevertheless, *BTN3A3* lacks a murine orthologue; valid *in-vivo* protein detection requires humanized mouse models, which was beyond the scope of the present study. It should be noted that molecular docking represents only an in-silico prediction and cannot serve as definitive proof of physical protein-drug interaction in biological systems.

**Figure 10:**
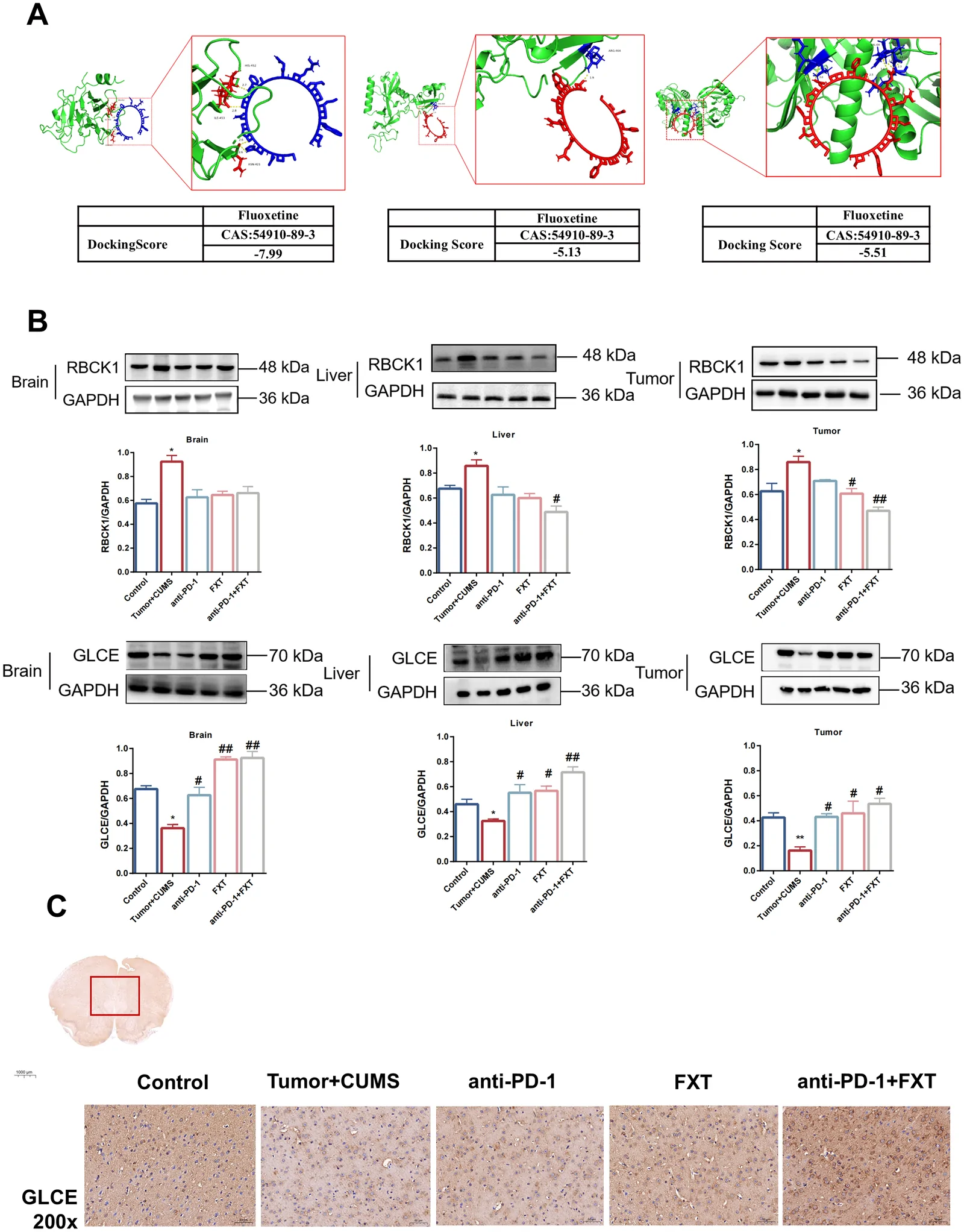
In silico binding prediction of fluoxetine against hub proteins and *in vivo* tissue expression detection of GLCE and RBCK1 in HCC-depression comorbid mice. **(A)** Molecular docking showing binding conformations and docking scores of fluoxetine with BTN3A3, GLCE and RBCK1. **(B)** Western blot bands and semi-quantitative statistics of GLCE and RBCK1 protein expression in mouse brain, liver and tumor tissues; GAPDH was used as internal reference. **(C)** Representative IHC staining images of GLCE in mouse prefrontal cortex tissues (200× magnification). Compared with the blank group, *p < 0.05 or ^**^p < 0.01; compared with the model group, ^#^*p* < 0.05 or ^##^*p* < 0.01. n=3.

Western blot was used to detect protein levels of GLCE and RBCK1 in mouse brain, liver and tumor tissues. Compared with the Tumor+CUMS model group, the model exhibited elevated RBCK1 protein expression and suppressed GLCE expression across all three tissues; fluoxetine monotherapy, anti-PD-1, and their combination partially or fully reversed these abnormal protein alterations (**Figure 10B**). Such coordinated protein changes implied that altered abundance of these two hub proteins correlates with HCC-depression comorbid pathological status, and drug intervention could rescue their dysregulated expression *in vivo*.

Immunohistochemical staining of prefrontal cortex tissue further verified markedly weakened GLCE positive signals in comorbid model mice, while combined fluoxetine plus anti-PD-1 treatment restored GLCE protein abundance (**Figure 10C**). Taken together, these in silico docking predictions together with *in-vivo* tissue expression data suggested that fluoxetine may interact with the hub proteins and modulate their protein abundance during HCC-depression comorbidity. Given the absence of mouse *BTN3A3* homologue, further functional assays including humanized animal models are required to confirm direct drug-target regulatory effects.

## 4 Discussion

This study systematically delineates the core shared immunometabolic axis driving hepatocellular carcinoma (HCC)-depression comorbidity centered on disrupted tryptophan-kynurenine signaling. Clinically, HCC patients suffer high depressive morbidity (54), yet unified molecular pathways and matched combinatorial therapeutic regimens linking the two disorders remain insufficiently characterized. Tryptophan metabolism acts as a central bridge between tumor immunity and neuropsychiatric homeostasis: under tumor burden and chronic stress (55), hepatic and systemic indoleamine 2,3-dioxygenase (IDO) and tryptophan 2,3-dioxygenase (TDO) are persistently activated, which accelerates tryptophan consumption and massive kynurenine accumulation (56, 57). Excess kynurenine derivatives polarize macrophages toward an immunosuppressive M2 phenotype, boost secretion of IL-10 and TGF-β, blunt anti-tumor CD8^^+^^T cell responses (58), and simultaneously cross the blood-brain barrier to disrupt astrocyte and neuron function, triggering anhedonia, social withdrawal and prolonged immobility (59). In the present work, we integrated two-sample Mendelian randomization (MR), genetic colocalization, multi-layer transcriptomics and targeted LC-MS/MS metabolomics alongside *in vivo* murine assays to dissect this axis. Unlike prior research relying solely on transcriptional enrichment inference, our combined transcript-metabolite dataset (**Figure 8**) provided absolute quantitative metabolic evidence verifying disturbed tryptophan-kynurenine flux in brain and peripheral circulation. Using a strict SNP.PP.H4 > 0.90 threshold, three hub genes (*GLCE*, *RBCK1*, *BTN3A3*) with consistent genetic correlation to both diseases were screened. Single-cell and spatial transcriptomics further mapped their cell-type-specific localization: *GLCE* predominates in hepatocytes and astrocytes, *RBCK1* enriches in vascular endothelial cells and excitatory neurons, and *BTN3A3* selectively expresses in T cells and microglia. Distinct tissue distribution patterns underpin their divergent regulatory roles in tumor and neuroinflammatory networks, jointly constructing a gene-tryptophan-kynurenine immune regulatory network underlying comorbid immune dysfunction.

With all three hub genes satisfying identical genetic colocalization criteria, their translational prospects require balanced differentiated interpretation. Molecular docking predicted fluoxetine bears the highest binding affinity for *BTN3A3*, yet this gene is primate-specific without functional murine orthologs (9). Conventional rodent models cannot support its *in vivo* protein detection or genetic manipulation, and existing single-cell datasets only link *BTN3A3* to γδ T cell activation, lacking direct association with systemic tryptophan metabolic reprogramming (9). In contrast, *GLCE* and *RBCK1* have complete mouse homologs and exert broader regulatory effects on immunometabolism. Transcription factor enrichment identified *E2F4* as the top upstream transcription factor of *GLCE* (**Figure 3D**), and multi-omic signatures linked both genes to NF-κB, IL6-JAK-STAT3 cascades and kynurenine metabolic reprogramming (60, 61). Western blot and IHC of mouse brain, liver and tumor tissues demonstrated consistent expression shifts: the Tumor+CUMS group exhibited elevated RBCK1 and reduced GLCE (62, 63), while fluoxetine combined with anti-PD-1 partially rescued these abnormal protein levels (**Figure 10**) (64). It is important to emphasize that in silico docking merely offers predictive binding clues, rather than definitive proof of direct drug-protein functional interaction; all descriptive language throughout the manuscript strictly distinguishes computational simulation from verified biological causality.

Building on the molecular features of the three hub genes, our animal data clarified the synergistic immunometabolic mechanism of fluoxetine plus anti-PD-1 against HCC-depression comorbidity. Monotherapy carries complementary limitations: anti-PD-1 restores exhausted tumor-infiltrating T cells with negligible antidepressant capacity, whereas fluoxetine relieves depressive behaviors yet cannot fully reverse tumor immune suppression (65). In HCC-CUMS mice, the combined regimen suppressed tumor proliferation, alleviated core depressive-like phenotypes, reduced peripheral CD206^^+^^ M2 macrophage proportions and lowered serum IL-10/TGF-β concentrations (**Figures 1**, **5**). Targeted LC-MS/MS further confirmed the combination normalized disordered L-tryptophan and L-kynurenine pools in brain and serum (**Figure 8**). Mechanistically, fluoxetine may modulate GLCE and RBCK1 to constrain M2 polarization and rectify abnormal kynurenine flux, while anti-PD-1 lifts checkpoint-mediated immune exhaustion, forming a synergistic circuit targeting shared tryptophan immune dysfunction. Notably, the absence of IDO1 inhibitor positive control groups prevents quantitative comparison of this regimen against classic tryptophan-targeted therapies (66), so all therapeutic efficacy descriptions avoid overstated claims of clinical superiority.

Despite the multi-omic analytical pipeline applied here, all bioinformatic outputs represent statistical correlation rather than confirmed causal relationships. All MR and colocalization analyses utilized European-ancestry FinnGen GWAS data exclusively, without cross-cohort validation in East Asian HBV-related HCC populations, meaning observed genetic links cannot be generalized to Chinese comorbid patients. Additionally, this study lacks cellular gain/loss-of-function models for *GLCE*, *RBCK1* and *BTN3A3*, as well as *in vivo* adenovirus/lentivirus genetic rescue experiments, leaving unvalidated whether these genes directly govern IDO/TDO activity or macrophage polarization. Furthermore, the proposed systemic brain-liver-tumor immune crosstalk hypothesis lacks serial circulating cytokine and metabolite measurements from peripheral blood (67), so speculative interpretations of inter-organ communication have been appropriately moderated.

There remain several aspects to be further refined in the current work, and systematic follow-up research directions are outlined accordingly. Genetically, all MR datasets used herein were derived from European populations without independent validation in East Asian HCC cohorts, which limits the generalizability of the observed genetic associations across distinct ethnic groups. At the cellular functional level, genetically modified cell lines and *in vivo* gene rescue models of hub genes have not yet been established, leaving the direct regulatory effects of *GLCE* and *RBCK1* on tryptophan immune signaling unconfirmed. In terms of animal modeling, subcutaneous tumor xenografts cannot fully recapitulate the intrinsic hepatic immune microenvironment of orthotopic HCC (68); IDO inhibitor groups were absent for cross-comparison of immunomodulatory potency among tryptophan-targeted interventions. For clinical translational exploration, matched tissue and peripheral biospecimens from patients with concurrent HCC and depression were not collected, restricting our capacity to evaluate the diagnostic biomarker performance of *GLCE* and *RBCK1* via ROC analysis (69). In silico binding simulations between fluoxetine and hub proteins lack supporting *in vitro* protein interaction data, and functional exploration of *BTN3A3* in conventional murine models is hindered due to its primate-specific feature. To address these unresolved issues, follow-up work will supplement multi-layer experimental evidence: East Asian GWAS cohorts will be incorporated to replicate the genetic signals identified in this study; *GLCE*/*RBCK1* modified cell lines and humanized *BTN3A3* mice will be generated for comprehensive cellular functional assays; orthotopic HCC-CUMS mouse models will be constructed with single-disease and IDO inhibitor control arms; matched clinical biosamples will be collected to assess biomarker efficacy by ROC curves; *in vitro* binding assays will be supplemented to verify predicted drug-protein interactions. Serial detection of circulating tryptophan metabolites and inflammatory cytokines will be performed to systematically characterize the liver-brain immunometabolic crosstalk axis (70), and preclinical safety evaluation of fluoxetine combined with anti-PD-1 will also be carried out to solidify translational evidence for this combinatorial regimen.

## 5 Conclusions

This multi-layer preclinical study integrated bioinformatic multi-omics profiling and murine *in vivo* assays to identify *GLCE*, *RBCK1* and *BTN3A3* as shared hub genes linking hepatocellular carcinoma and depression through dysregulated tryptophan-kynurenine immunometabolic signaling. Mechanistic animal data further demonstrated that combined fluoxetine and anti-PD-1 treatment yields synergistic anti-tumor and antidepressant efficacy. This combinatorial regimen remodels M2 macrophage-enriched immunosuppressive microenvironments and recovers disrupted systemic tryptophan metabolic balance, offering a promising preclinical intervention strategy for HCC-depression comorbidity. Although the present work constructs an integrated gene-metabolite-immune regulatory network underlying comorbid immune disturbance, several experimental constraints remain unaddressed. Follow-up cellular functional assays, humanized animal models and clinical cohort analyses are required to fully validate the molecular regulatory mechanisms and translational applicability of the three hub genes.

## Data Availability

The original contributions presented in the study are included in the article/**Supplementary Material**. Further inquiries can be directed to the corresponding authors.
