# Supplementary material for: Shared immune perturbations in hepatocellular carcinoma-depression comorbidity and synergistic immunotherapeutic effects of fluoxetine combined with anti-PD-1
Source: Front Immunol. 2026 Sep 25;17:1909503. doi: 10.3389/fimmu.2026.1909503 (PMC13649994; doi:10.3389/fimmu.2026.1909503)
Supplement: Supplementary file 1 [file DataSheet1.pdf]

Supplementary Information

Supplementary Tables S1-S5

Table S1 CUMS Manipulation Protocols

Stressor	Operation protocol
5 min ice-water bath	Place mice in 4 ℃ ice- water for swimming for 5 min
24 h food-water deprivation	Remove food and water supplies from the cage for 24 h
24 h wet bedding	Moisten bedding with 200 mL water per 100 g bedding
24 h light- dark reversal	Cover cages with black shading cloth during daytime; illuminate cages with LED lights at night for 24 h
10 min tail clamping	Wrap a tail clip with cotton gauze, clamp 1 cm from the base of the mouse tail for 10 min
24 h crowding housing	House 12- 15 mice in a single cage for 24 h
30 min horizontal shaking	Place cages on a horizontal shaker at 150 rpm for30 min
24 h empty cage housing	Keep mice in cages without bedding for 24 h
24 h cage tilting	Maintain cages at a 45° tilt angle for 24 h
3 h restraint	Restrain mice in perforated and ventilated 50 mL centrifuge tubes for 3 h
4 h noise exposure	Expose mice to white noise at 85 dB for 4 h

Table S2 CUMS Stressor Schedule

Stressor	Monday	Tuesday	Wednesday	Thursday	Friday	Saturday	Sunday
5 min ice- water bath	√						√
24 h food- water deprivation		√					
24 h wet bedding			√				
24 h light- dark reversal				√			
10 min tail clamping					√		
24 h crowding housing					√	√	
30 min horizontal shaking							√
24 h empty- cage housing							
24 h cage tilting		√		√			
3 h restraint			√				
4 h noise exposure	√					√	

Table S3: Summary of major software, *R* packages, database versions, and RRIDs

Category	Software/Package/ Database	Purpose	Version	RRID
Statistical Platform	R / RStudio	All statistics and graphics	4.3.0 / 2025.05.0+496	SCR_001905 / SCR_000432
Bioconductor	Bioconductor	Package management framework	3.18	SCR_006442
MR/co-localization	TwoSampleMR;coloc	Two-sample MR; co-localization	0.6.22 / 5.2.3	—
Single cell	Seurat	QC, Dimensionality reduction, clustering, and annotation	4.3.0	SCR_016341
Single cell	harmony	Batch effect correction	1.1.0	SCR_022206
Single cell	DoubletFinder	Doublet filtering	2.0.4	SCR_018771
Single cell	sctransform;AUCell	Normalization; pathway activity scoring	0.4.1 / 1.24.0	—
Spatial transcriptomics	spacexr (RCTD)	Spatial deconvolution	2.2.1	—
Spatial transcriptomics	mistyR;progeny/decoupleR	Spatial interaction; pathway activity scoring	1.16.0 / 1.24.0 / 2.8.0	—
Enrichment analysis	clusterProfiler	GSEA/GO/KEGG enrichment	4.17.0	SCR_016884
Enrichment analysis	GSVA;GSEABase	GSVA/ssGSEA scoring	1.50.5 / 1.64.0	SCR_021058
Differential/Linear Model	limma	Differential-expression analysis and gene ranking	3.58.1	SCR_010943
Visualization	ComplexHeatmap;ggplot2;pheatmap;corrplot	Heatmaps and general plotting	2.18.0 / 3.5.2 / 1.0.13 / 0.95	SCR_017171 / SCR_014601 / —
Immune deconvolution	CIBERSORT	Deconvolution of 22 immune cell subtypes	v1.03 (R script)	SCR_016955
Correlation analysis	Hmisc(rcorr)	Pearson correlation	5.2-3	—
Network	Cytoscape	miRNA-immune interaction network visualization	3.10.3	SCR_003032
Molecular docking	AutoDock;PyMOL	Docking; visualization	—	SCR_012746/ SCR_000305

Category	Software/Package/ Database	Purpose	Version	RRID
Database	TCGA/GDC;GEO; eQTLGen;FinnGen ;MSigDB;Cell Ranger	Expression/genetics/annotation data	MSigDB v7.0;Cell Ranger not directly used	SCR_003193/ SCR_014517/ SCR_005012/ —/SCR_016863/ SCR_017344

Table S4 Summary of bulk transcriptome dataset information

Dataset	Disease/organization	Data type	Sample size	Platform/ Source	Key points for handling
TCGA-LIHC	HCC vs. adjacent non-tumor liver tissue	RNA - seq (gene level)	424 cases (374 tumors + 50 non-tumor)	GDC unified pipeline	$\log_2(x+1)$; No batch-effect correction; No additional filtering for low-expressed genes
GSE80655	Depression vs Control(post-mortem brain tissue)	Microarray (Illumina HumanHT-12 v4.0)	139 cases (69 MDD+70 controls)	GPL11154	GPL11154 probe-symbol mapping deduplication; $\log_2(x+1)$; No batch-effect correction

Table S5. Summary of bioinformatic and statistical analyses

Analysis Module	Software/Method	Statistical test	Multiple comparison correction/threshold
Two-sample MR	TwoSampleMR	IVW (meta-analysis), MR-Egger,Weighted-median, weighted-mode; Single SNP using Wald ratio; Cochran's Q Heterogeneity Test; Leave-one-out sensitivity analysis	IV screening with $P< 5\times10^{-8}$; LD clumping $R^2<0.001$(10,000 kb); $F>10$; IVW $P<0.05$ is considered positive
Co-localization	coloc	Co-localization posterior probability	PP.H4>0.90
GSEA	clusterProfiler (gseKEGG/gseGO)	Median grouping of key genes; Limma logFC sorting; Permutation test	Adjusted $P<0.05$(default BH)
GSVA	GSVA package (kcdf=Gaussian)	Group-level differences in pathway activity:limma (empirical Bayes)	Benjamini -Hochberg correction, $P<0.05$
Immune infiltration	CIBERSORT (LM22)	Support vector regression deconvolution; 100 permutations; quantile normalization	Deconvolution $P<0.05$

Analysis Module	Software/Method	Statistical test	Multiple comparison correction/threshold
Correlation analysis	Hmisc::rcorr (*Pearson*)	*Pearson* correlation	P<0.05, P<0.01, P<0.001 Graded significance labeling
Single cell scoring	AUCell; GSVA ssGSEA (kcdf=Gaussian,abs.ranking)	Pathway activity score	—
Spatial deconvolution/interaction/path	RCTD (spacexr); MISTy (mistyR); PROGENy (progeny)	Deconvolution weight;mutual contribution; pathway activity	—
Molecular docking	AutoDock	9 docking attempts, take the lowest binding energy conformation	Binding energy (kcal/mol)
Overall	R 4.3.0	All statistical tests were two-tailed	P<0.05 was considered statistically significant

Supplementary Fig. S1

Gene	Exposure	Method	Liver Cancer(LC)		Depression(Dep)
BTN3A3	eq tl-a-ENSG00000111801	MR Egger	Q	1.664	3.645
			Q df	6.000	6.000
			Q pval	0.948	0.725
		Inverse variance weighted	Q	2.254	6.825
			Q_df	7.000	7.000
			Q pval	0.944	0.447
DRD4	eq tl-a-ENSG00000069696	MR Egger	Q	0.377	2.661
			Q df	1.000	1.000
			Q pval	0.539	0.103
		Inverse variance weighted	Q	1.570	2.798
			Q_df	2.000	2.000
			Q pval	0.456	0.247
FPGT	eq tl-a-ENSG00000254685	MR Egger	Q	0.482	1.309
			Q_df	1.000	1.000
			Q pval	0.487	0.253
		Inverse variance weighted	Q	0.939	2.398
			Q df	2.000	2.000
			Q pval	0.625	0.302
GLCE	eq tl-a-ENSG00000138604	MR Egger	Q	0.025	2.795
			Q_df	2.000	2.000
			Q pval	0.988	0.247
		Inverse variance weighted	Q	1.759	4.438
			Q df	3.000	3.000
			Q pval	0.624	0.218
RAC1	eq tl-a-ENSG00000136238	MR Egger	Q	1.038	0.270
			Q_df	1.000	1.000
			Q pval	0.308	0.603
		Inverse variance weighted	Q	1.113	0.794
			Q_df	2.000	2.000
			Q pval	0.573	0.672
RBCK1	eq tl-a-ENSG00000125826	MR Egger	Q	2.554	0.420
			Q_df	2.000	2.000
			Q pval	0.279	0.811
		Inverse variance weighted	Q	2.809	2.948
			Q_df	3.000	3.000
			Q pval	0.422	0.400
VPS33B	eq tl-a-ENSG00000184056	MR Egger	Q	2.111	0.016
			Q df	1.000	1.000
			Q pval	0.146	0.899
		Inverse variance weighted	Q	2.465	1.742
			Q_df	2.000	2.000
			Q pval	0.292	0.419

Supplementary Fig. S1 Mendelian randomization (MR) results for genes in liver cancer and depression, using MR Egger and inverse variance weighted methods.

Supplementary Fig. S2

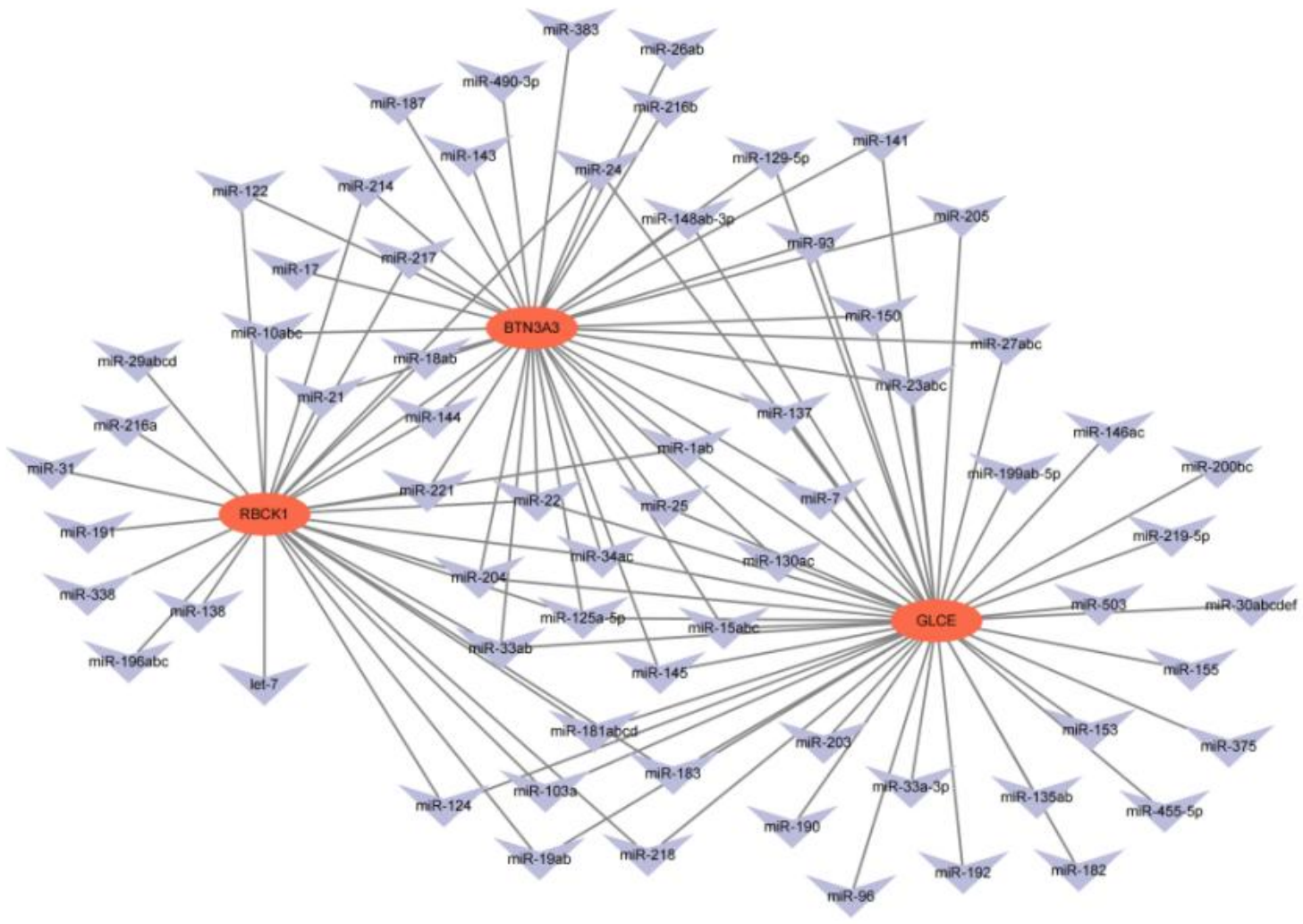

Supplementary Fig. S2 Network of key genes (*RBCK1*, *BTN3A3*, *GLCE*) and their associated miRNAs. Key genes were reverse-predicted via the miRcode database, yielding 67 miRNAs and 110 mRNA-miRNA pairs in total, which were visualized using Cytoscape.

Supplementary Fig. S3

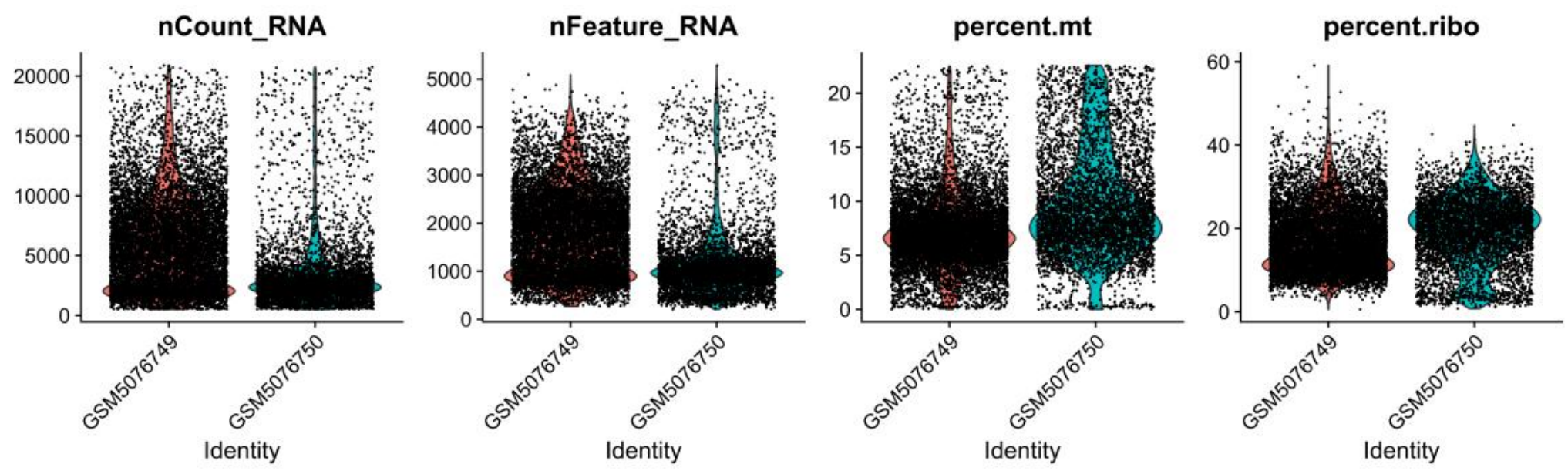

Supplementary Fig. S3. The distribution of nCount_RNA, nFeature_RNA, percent.mt, and percent.ribo across samples GSM5076749 and GSM5076750 is shown by violin plots in liver cancer.

Supplementary Fig. S4

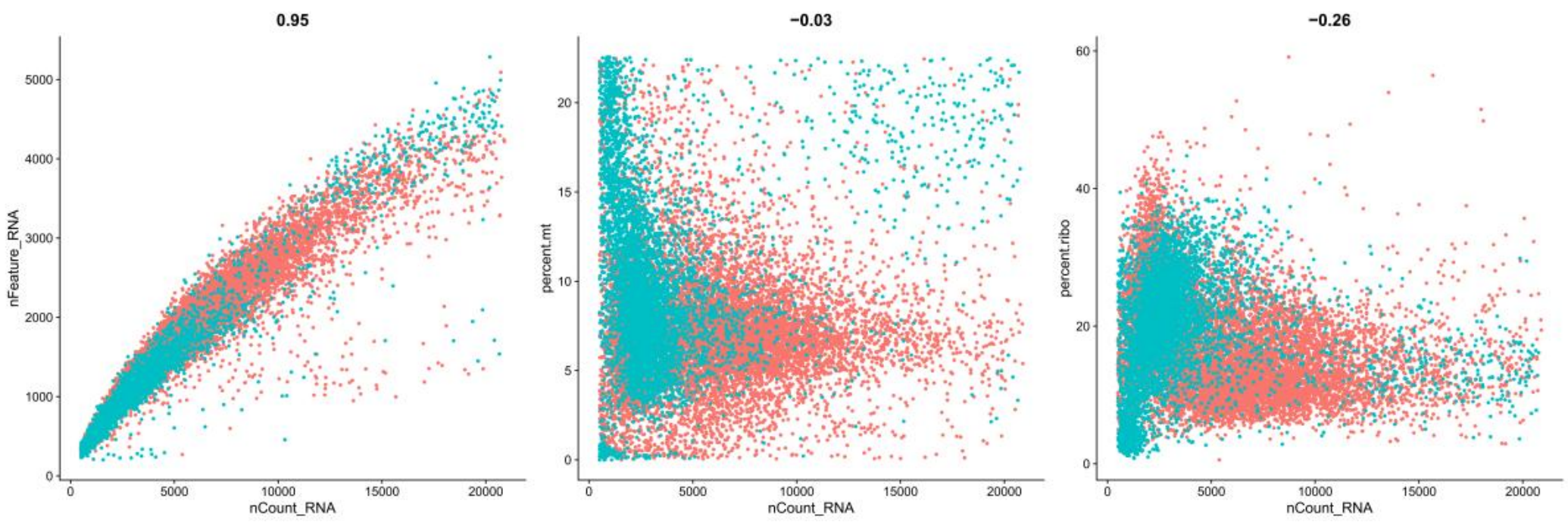

Supplementary Fig. S4. Correlations of nFeature_RNA with nCount_RNA, percent.mt with nCount_RNA, and percent.ribo with nCount_RNA are shown by scatter plots

Supplementary Fig. S5

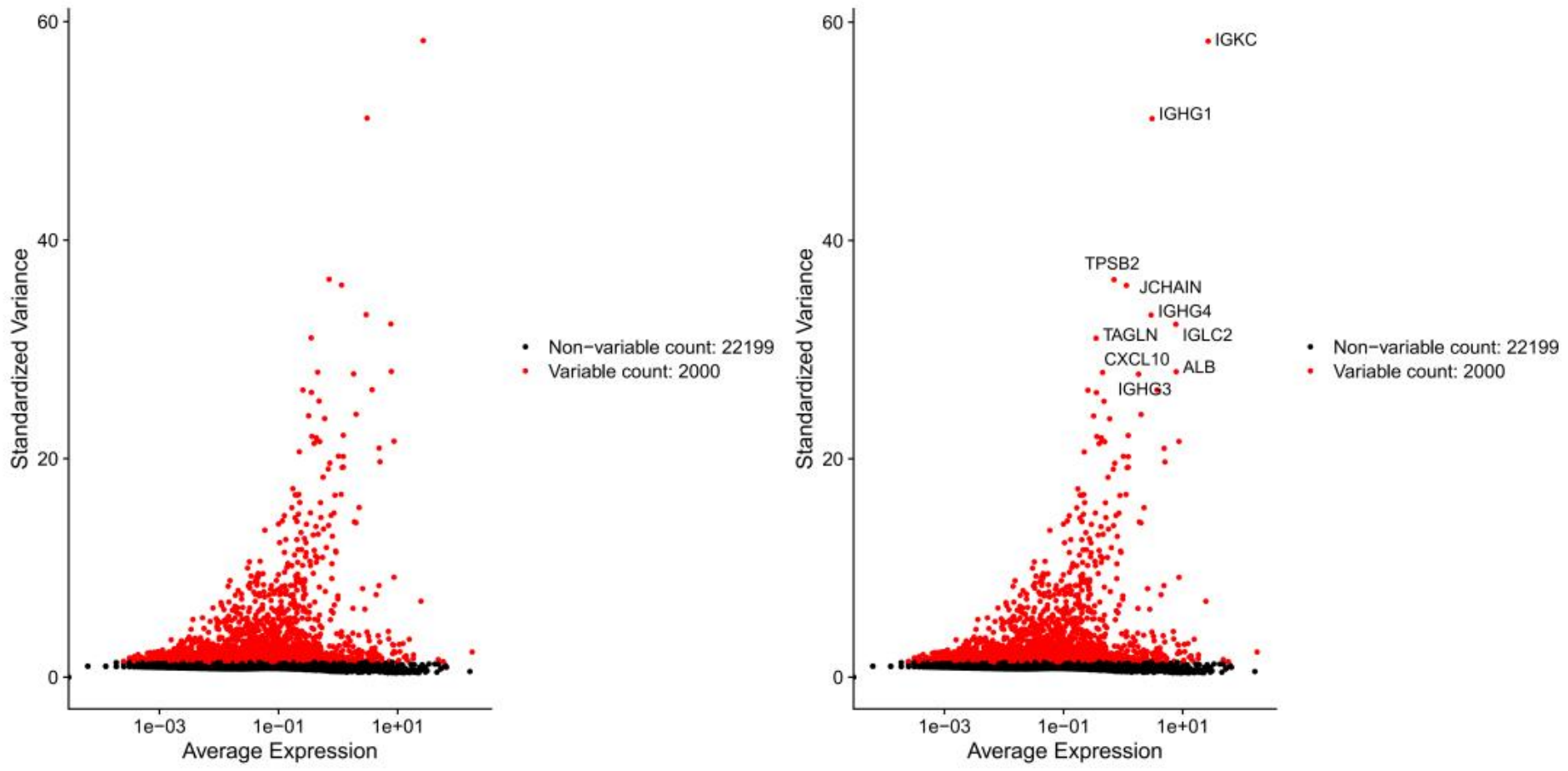

Supplementary Fig. S5. The selection of variable and non - variable genes, with their standardized variance and average expression, is shown by scatter plots.

Supplementary Fig. S6-S7

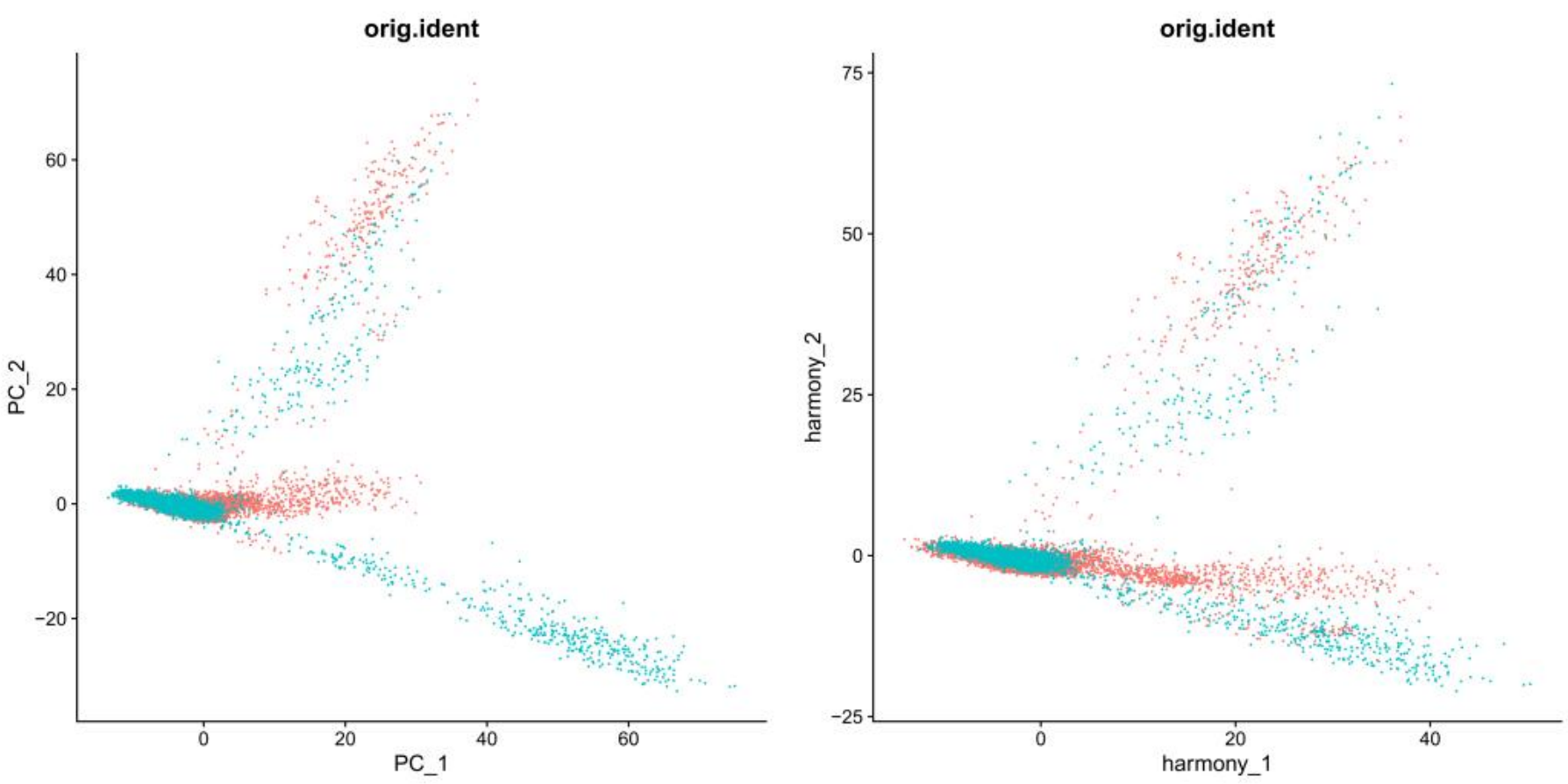

Supplementary Fig. S6-7. The distribution of cells in PC (principal component) space and

Harmony - adjusted space is shown by scatter plots.

Supplementary Fig. S8

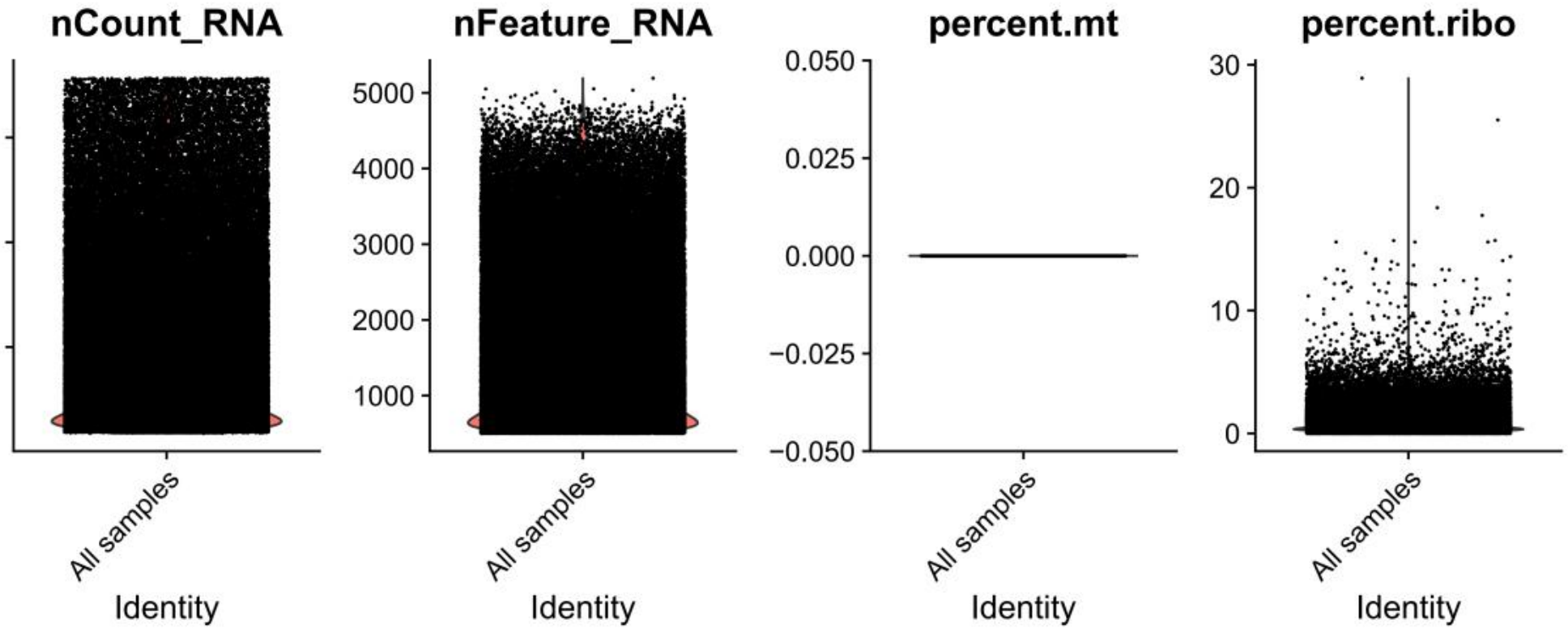

Supplementary Fig. S8. The distribution of nCount_RNA, nFeature_RNA, percent.mt, and percent.ribo across all samples is shown by violin plots in depression.

Supplementary Fig. S9

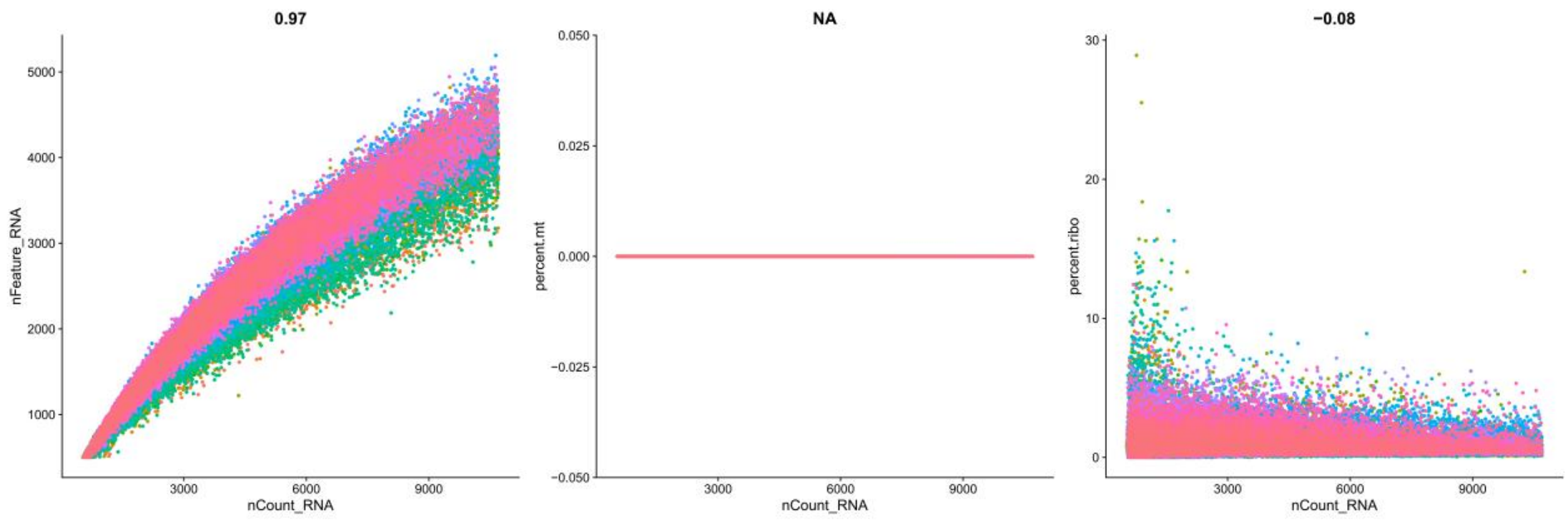

Supplementary Fig. S9 Correlations of nFeature_RNA, percent.mt, and percent.ribo with nCount_RNA are shown by scatter plots.

Supplementary Fig. S10

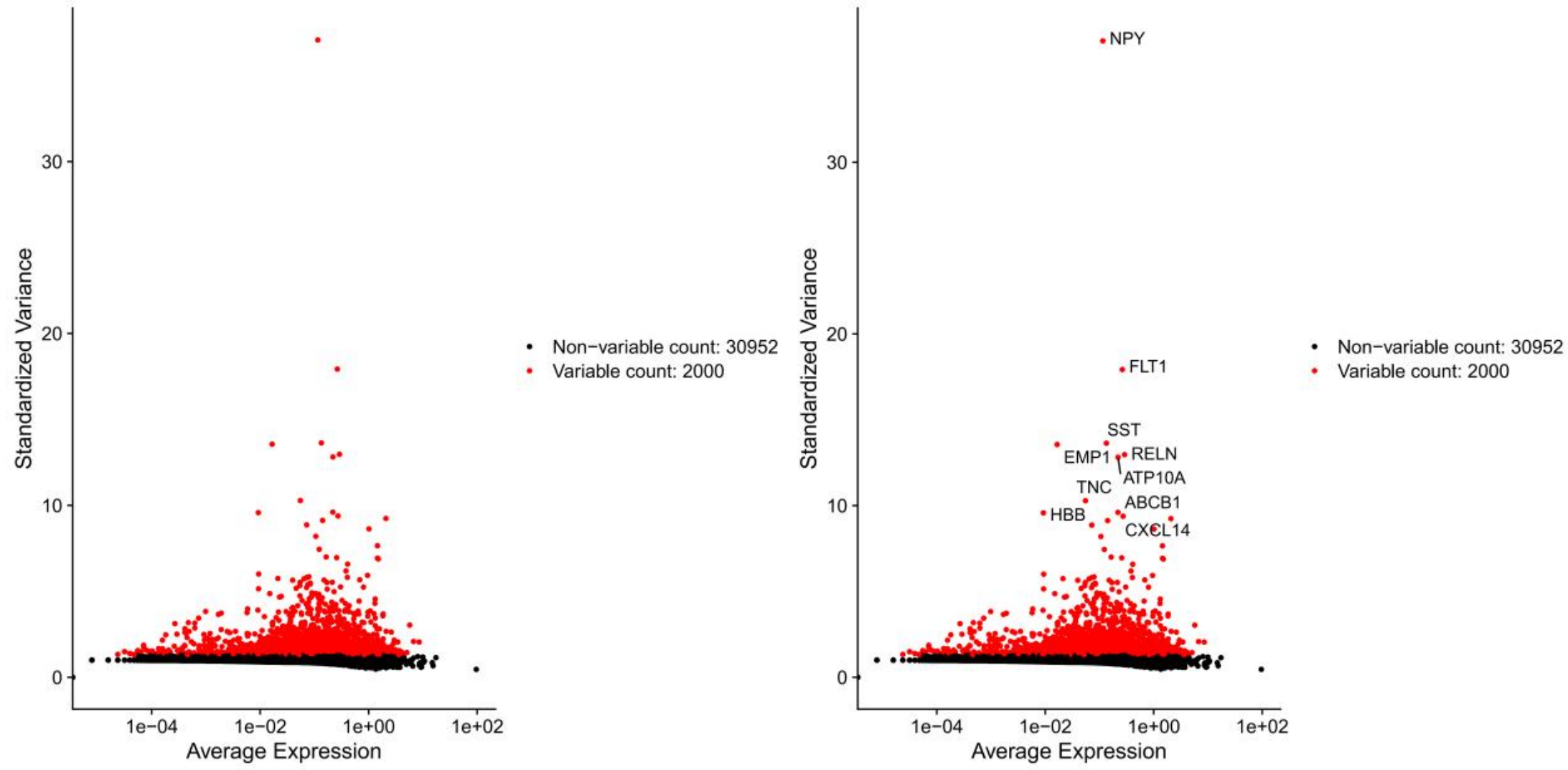

Supplementary Fig. S10. The selection of variable and non - variable genes, along with their standardized variance and average expression is shown by scatter plots.

Supplementary Fig. S11

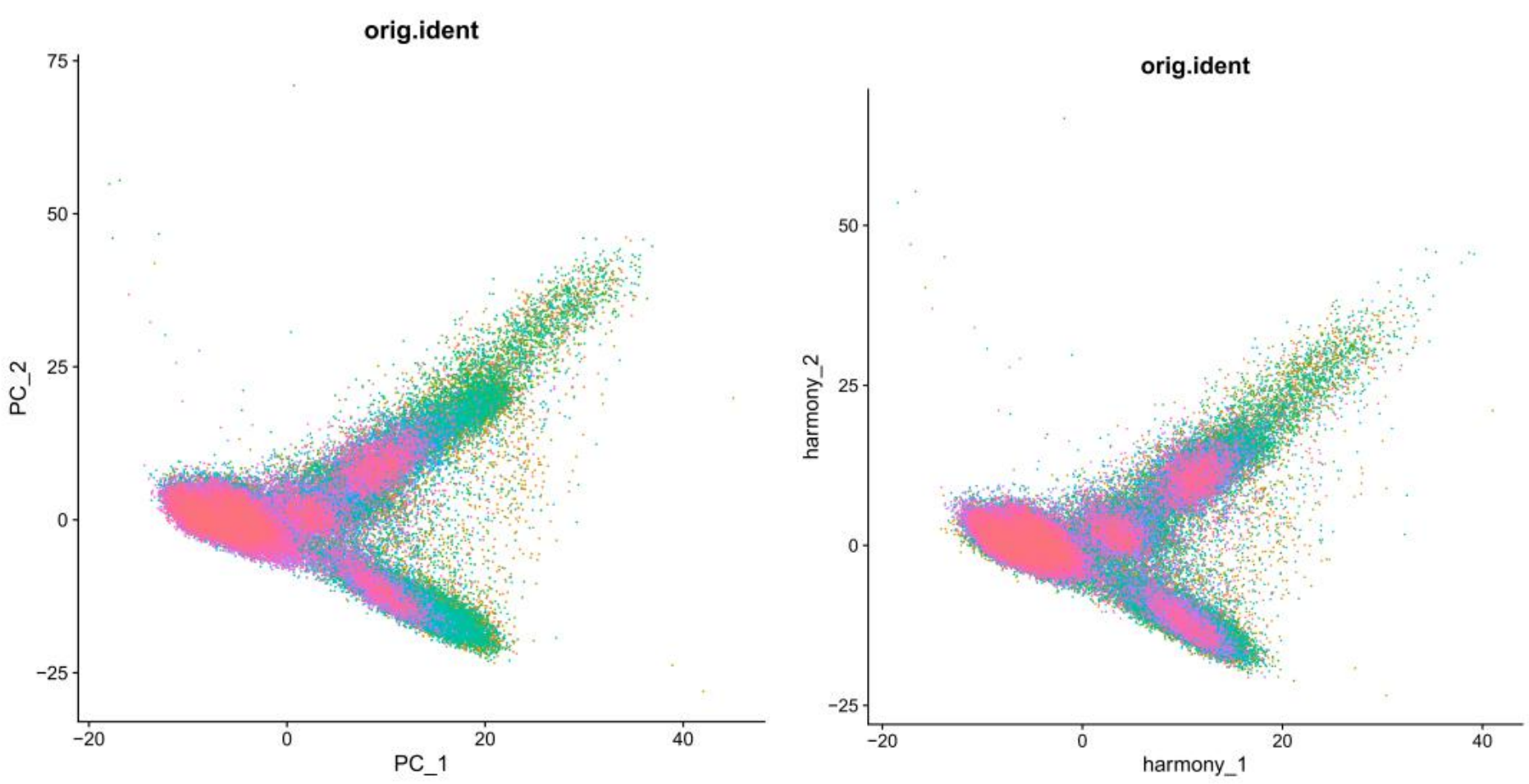

Supplementary Fig. S11. The distribution of cells in PC space and Harmony - adjusted space, colored by is shown by scatter plots.

Supplementary Fig. S12

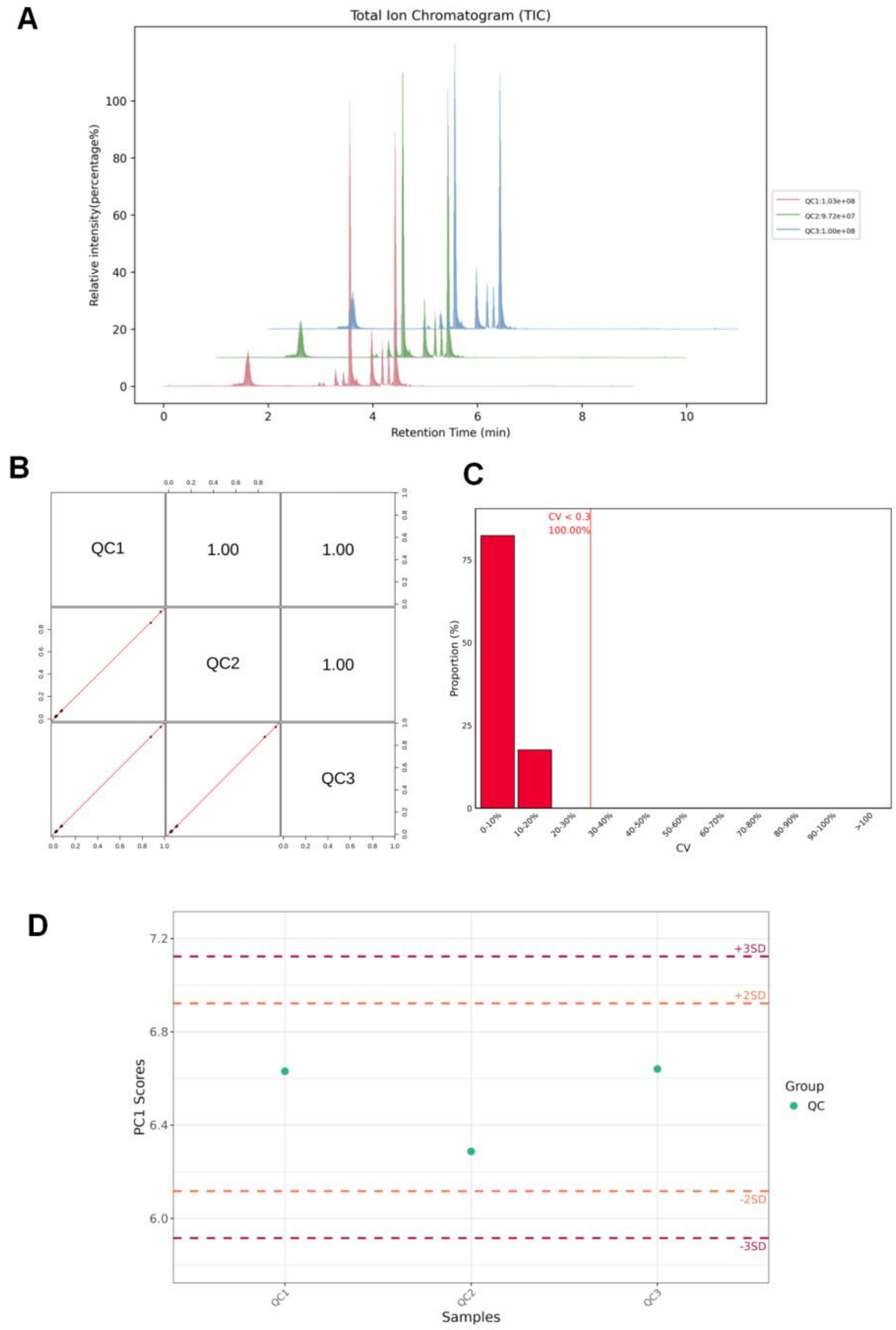

Supplementary Figure S12. Quality control of tryptophan-targeted LC-MS metabolomics. (A) Overlaid total- ion chromatogram (TIC) of QC samples. (B) Correlation analysis among QC samples. (C) CV distribution of metabolites detected in QC samples. (D) PCA- based quality-control assessment of QC samples.
